# Intranasal delivery of human umbilical cord Wharton's jelly mesenchymal stromal cells restores lung alveolarization and vascularization in experimental bronchopulmonary dysplasia

**DOI:** 10.1002/sctm.18-0273

**Published:** 2019-11-27

**Authors:** Alvaro Moreira, Caitlyn Winter, Jooby Joy, Lauryn Winter, Maxwell Jones, Michelle Noronha, Melissa Porter, Kayla Quim, Alexis Corral, Yasmeen Alayli, Tyrelle Seno, Shamimunisa Mustafa, Peter Hornsby, Sunil Ahuja

**Affiliations:** ^1^ Department of Pediatrics, Cellular and Integrative Physiology University of Texas Health Science Center San Antonio (UTHSCSA) San Antonio Texas; ^2^ Microbiology and Immunology University of Texas Health Science Center San Antonio (UTHSCSA) San Antonio Texas

**Keywords:** bronchopulmonary dysplasia, intranasal delivery, mesenchymal stromal cell, neonate, umbilical cord

## Abstract

Bronchopulmonary dysplasia (BPD) is a devastating lung condition that develops in premature newborns exposed to prolonged mechanical ventilation and supplemental oxygen. Significant morbidity and mortality are associated with this costly disease and effective therapies are limited. Mesenchymal stem/stromal cells (MSCs) are multipotent cells that can repair injured tissue by secreting paracrine factors known to restore the function and integrity of injured lung epithelium and endothelium. Most preclinical studies showing therapeutic efficacy of MSCs for BPD are administered either intratracheally or intravenously. The purpose of this study was to examine the feasibility and effectiveness of human cord tissue‐derived MSC administration given via the intranasal route. Human umbilical cord tissue MSCs were isolated, characterized, and given intranasally (500 000 cells per 20 μL) to a hyperoxia‐induced rat model of BPD. Lung alveolarization, vascularization, and pulmonary vascular remodeling were restored in animals receiving MSC treatment. Gene and protein analysis suggest the beneficial effects of MSCs were attributed, in part, to a concerted effort targeting angiogenesis, immunomodulation, wound healing, and cell survival. These findings are clinically significant, as neonates who develop BPD have altered alveolar development, decreased pulmonary vascularization and chronic inflammation, all resulting in impaired tissue healing. Our study is the first to report the intranasal delivery of umbilical cord Wharton's jelly MSCs in experimental BPD is feasible, noninvasive, and an effective route that may bear clinical applicability.

## Graphical abstract

Intranasal delivery of human umbilical cord mesenchymal stromal cells improves lung growth and development in a rat model mimicking neonatal lung disease.

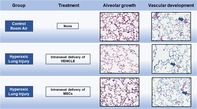

**Significance statement**

Bronchopulmonary dysplasia (BPD) is the most common cause of morbidity and mortality in extremely premature neonates. Unfortunately, current therapies for BPD are limited. Preclinical studies have shown that mesenchymal stem cell (MSC) treatment can restore alveolar growth, enhance vascular development, and stimulate tissue repair. Most of these studies have administered the cells via the intravenous/tracheal route. Results of this study show, for the first time to the authors' knowledge, that the intranasal delivery of MSCs for BPD is effective in restoring lung alveolar growth and vascular development. Importantly, this study provides evidence that this noninvasive approach may be given separately or as an adjunct/alternate to other routes.

## 1 INTRODUCTION

Preterm birth is a major health concern affecting approximately 12% of all deliveries in the United States and costing over $26 billion every year.^1^ The most common cause of morbidity in premature neonates is a devastating lung disease known as bronchopulmonary dysplasia (BPD).^2^ BPD is characterized by a simplification of lung development following long‐term exposure to mechanical ventilation and supplemental oxygen, both essential treatments for extremely preterm neonates.^3^, ^4^ Neonates who develop BPD are at increased risk for cognitive impairment, frequent hospitalizations, and lifelong cardiopulmonary disease.^5^, ^6^, ^7^ The pathophysiology of BPD is complex and encompasses multiple processes including inflammation, oxidative stress, abnormal vasculogenesis, and impaired lung repair.^8^, ^9^, ^10^ Effective treatment options are limited, leaving a vulnerable patient population with an unmet need to develop novel therapies.

Regenerative medicine is an emerging field that seeks to remedy complex disease states by “stimulating previously irreparable organs to heal themselves.”^11^, ^12^ Mesenchymal stem/stromal cells (MSCs) are regenerative cells that can repair injured tissue by secreting multiple paracrine factors that can immunomodulate and restore function and lung epithelial/endothelial integrity.^13^, ^14^ Numerous preclinical studies have shown that treatment with MSCs can alleviate neonatal lung injury in animal models mimicking BPD.^15^ Most of these rodent studies have focused on administering the cells via an intratracheal or intravenous route.^16^, ^17^, ^18^, ^19^ To date, the optimal number of administrations and route of MSC delivery to treat BPD are unknown. Therefore, the primary goal of our study was twofold: (a) determine feasibility of intranasal delivery of human umbilical cord tissue MSCs, a clinically relevant and noninvasive approach to deliver multiple doses, in neonatal rat pups, and (b) examine whether intranasal delivery of MSCs attenuates lung injury in experimental BPD.

## 2 MATERIALS AND METHODS

### 2.1 Cell culture and MSC preparation

Umbilical cord Wharton's jelly tissue was collected from a healthy term human newborn. After tissue enzymatic digestion, cells were expanded on culture dishes under standard conditions. Wharton's jelly cells met the minimum criteria for MSCs, as described in our previous publication.^20^

Preparation of MSCs was achieved as follows, 5 × 10^5^ cells were seeded onto 100 mm tissue culture dishes and grown under standard conditions with media changes every 2‐3 days. At 24 hours prior to administration, cells were checked for confluency (80%‐90%) and the media was changed for the last time. On the day of administration, the media was removed from the dishes and the cells were washed twice with phosphate buffered saline. TrypLE Express enzyme (Thermo Fisher Scientific, Waltham, MA) was added to the dishes for 5 minutes to allow the cells to detach and an equal volume of αMEM (minimum essential medium) was added to the dishes to deactivate the TrypLE. The cell suspension was transferred to a conical tube, counted, and distributed into 20 μL aliquots containing 5 × 10^5^ cells and phosphate buffered saline (PBS, Sigma‐Aldrich). We derived our dose based on the systematic review and meta‐analysis conducted by Augustine et al (highest standardized mean difference favoring MSC treatment for alveolarization).^21^ Cells were kept on ice until administration. Immediately before intranasal delivery, the cell suspension was warmed to body temperature to avoid animal stress. All cells in the experiments were seeded at passage three.

### 2.2 Animal maintenance

All animal protocols were approved by The University of Texas Health Science Center (UTHSCSA) Institutional Animal Care and Use Committee. The animals received care in compliance with the *Guide for the Care and Use of Laboratory Animals* and experiments were carried out in compliance with the Helsinki Declaration.

Timed pregnant female Sprague‐Dawley rats were obtained from Charles River Laboratories at E14‐E15 days of gestation. Animals were singly housed with 12‐hour light/dark cycles, standard rodent laboratory diet and water was provided ad libitum. Dams were provided with nesting material at E18‐E19 onwards and received DietGels (Clear H_2_O, Portland, ME) with cage changes (every 48 hours).

**Figure 1:**
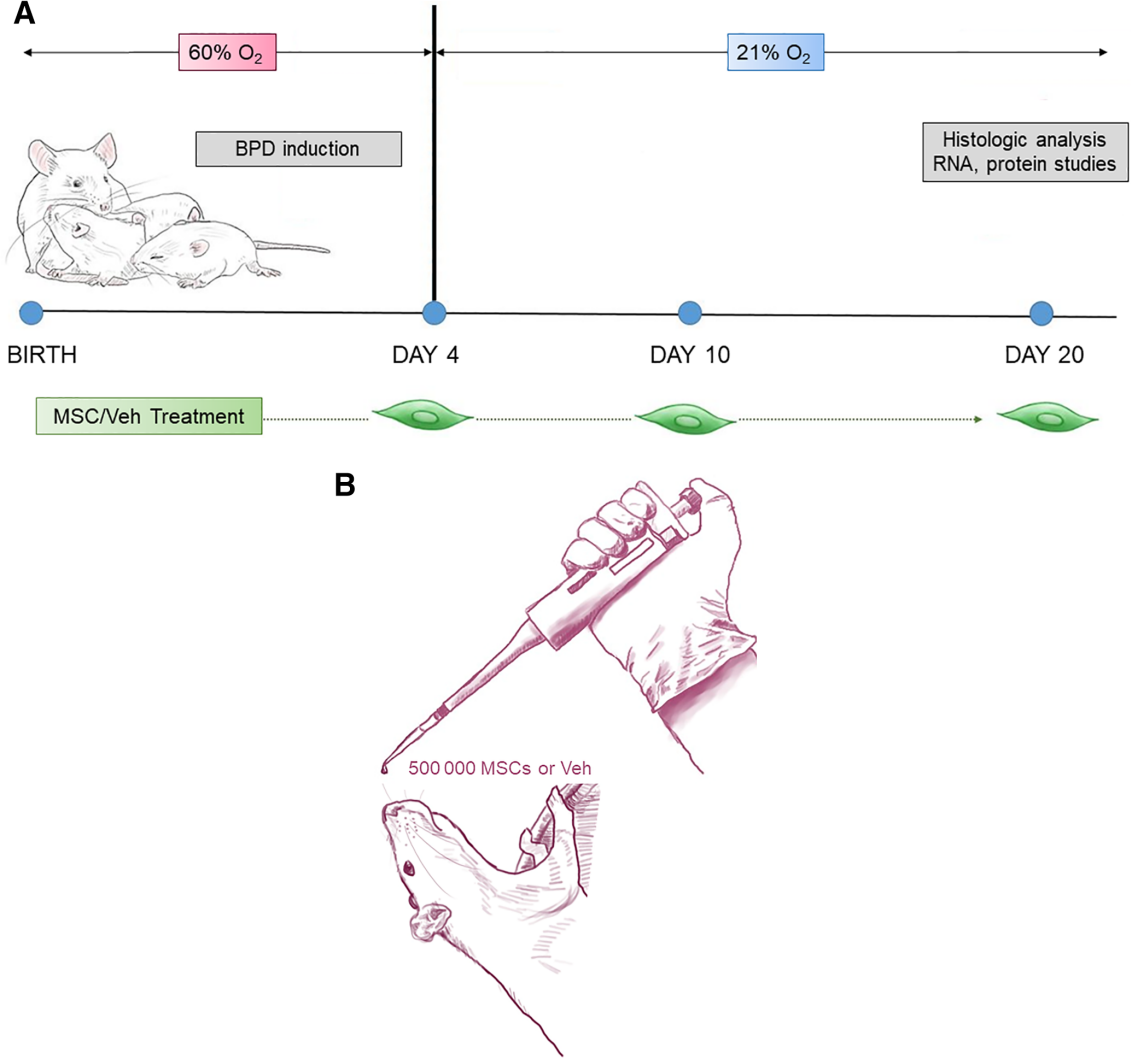
Experimental design: A, Newborn rats were exposed to 60% O_2_ for 4 days to induce bronchopulmonary dysplasia (BPD). BPD animals were compared to rats that were maintained in room air (RA, 21% O_2_). On days 4, 10, and 20, BPD treatment animals received either mesenchymal stromal cell (MSC) or vehicle (Veh). Outcomes were performed on days 20‐21. B, Schematic representation of intranasal delivery to Sprague‐Dawley rat pups. As noted, the animals were in an erect position with their necks slightly extended to facilitate delivery to the lungs

On postnatal day 4, newborn rat pups were randomly assigned into four groups: (a) room air (RA), (b) BPD, (c) BPD treated with αMEM as a vehicle (BPD + Veh), and (d) BPD treated with mesenchymal stromal cells (BPD + MSCs). RA animals were survived at normoxia (21% O_2_) for 21 days. The remaining BPD groups were exposed to 4 days of continuous hyperoxia (60%) using a BioSpherix animal housing chamber (BioSpherix Ltd, Lacona, NY).^22^, ^23^, ^24^, ^25^ Following a moderate BPD induction, animals were housed the remainder of the 3 weeks in normoxia. Pups were marked using toe tattoos specific to each treatment group.^26^ BPD rats received iterative treatments of vehicle or MSCs, on days 4, 10, and 20. Body weights were measured on each treatment day. Figure 1A summarizes the experimental design.

### 2.3 Intranasal delivery of MSCs or vehicle

Intranasal delivery of cells or vehicle was achieved using a modified version of the methods as described by Hanson et al.^27^, ^28^ Briefly, neonatal rats were held in the nondominant hand, with the body of the animal supported by the thumb and base of the palm and the head gently immobilized between the first and second finger. For older animals, the same support was used, but the head was immobilized between the thumb under the chin and the first and second fingers just behind the ears. To encourage the treatment to travel to the lungs rather than the CNS, animals were oriented vertically, with the coronal plane perpendicular to the ground and the neck upright and extended.^29^ Therapies were administered using a 2‐20 μL micropipettor with extra‐long gel loading tips (Fisher Scientific, Waltham, MA) to facilitate droplet formation (refer to Figure 1B). The total instillation volume (20 μL) was administered over a 5‐minute period; this allowed for ample recovery time and aliquoted delivery to the animals.

### 2.4 Tissue processing

Animals were euthanized by CO_2_ exposure followed by thoracotomy. Carcasses were kept on ice until tissue processing was completed. Lungs were excised, and the right lung was ligated, removed, and flash frozen in liquid nitrogen for protein and RNA analysis. The left lung was inflation fixed with 4% paraformaldehyde instilled through the trachea at 20 cm pressure for 2 minutes. After 24 hours, tissues were transferred to 70% ethanol prior to histologic analysis. Lungs were cut into four levels and oriented with alternating surfaces exposed prior to creation of 5 μm paraffin embedded sections. The heart was excised and the apex flash frozen, the remaining heart tissue was fixed in 10% neutral buffered formalin. The atria of the hearts were removed, and the remaining tissue oriented such that transverse sections of the ventricles could be obtained after paraffin embedding. All histological sectioning and staining were performed by UTHSCSA's Pathology Laboratory.

Lung slides were stained with hematoxylin and eosin stain, von Willebrand Factor, Masson's Trichrome, and α‐smooth muscle actin. Digital slides were created using the Aperio CS2 Digital Pathology Slide Scanner (Leica Biosystems, Wetzlar, Germany). Measurements were made using the following software: Aperio ImageScope software version 12.3.3, Pathomation (Belgium), MIPCloud (Iran), Image J, and Digimizer (Belgium).

### 2.5 Detection of human umbilical cord MSCs in rat lung tissue

Five micron‐thick sections were cut from paraffin‐embedded lung tissue, deparaffinized, and stained with a monoclonal antibody specific to intact human mitochondria (MAB 1273, EMB Millipore, Burlington, VA) as described in Allard et al.^30^ All immunohistochemical staining was performed by the Histology and Immunohistochemistry Laboratory at UTHSCSA. Slide images were obtained at 20x magnification using Aperio slide scanning system and ImageScope software.

### 2.6 Lung morphometry

#### 2.6.1 Alveolarization

To obtain a comprehensive assessment of the lungs, we measured mean linear intercepts in a total of 270 random fields (we evaluated 3 random lung sections, 30 random fields, and 3 standardized measurements per field) on each animal.

#### 2.6.2 Alveolar tissue distribution

To discern airspace enlargement, we adapted the techniques described by Jacob et al.^31^ We randomly obtained 30 images per lung (10 images per 3 lung sections) that avoided major airways and vasculature. We uploaded the digital pictures onto MIPCloud for processing. The pictures covered a field of 727 μm × 482 μm, while the threshold (200) and region of interest remained constant (area spanning 235 μm × 166 μm). Supplementary Figure S1 portrays a sample image used to calculate alveolar tissue distribution.^31^

#### 2.6.3 Vasculogenesis

Pulmonary vessels were point counted on H&E stained lung sections measuring 727 μm × 482 μm and imaged at ×20 magnification. Nine viewing lung fields were randomly created for each animal. The total number of vessels was then averaged over the number of viewing fields analyzed to generate the presented values (vessels/viewing field). Findings were confirmed by randomly choosing four animal lungs per group (save BPD + Veh) for manual counting of von Willebrand factor stained vessels. Counts were done in 20 randomly selected lung fields measuring an area of 423 μm × 530 μm at a ×20 magnification.

#### 2.6.4 Vascular remodeling/muscularization

External diameters of pulmonary vessels ranging from 50‐200 μm were analyzed for medial wall thickness (MWT). The following equation was used to calculate MWT: ((External diameter ‐ Internal diameter) / (External diameter)) × 100.^32^ A total of five random pulmonary vessels per lung were analyzed in all animals. For each vessel, wall thickness was measured along the shortest length of vessel diameter. Digimizer was used to measure the medial wall thickness on digital images at ×20 magnification.

Immunostaining for α‐smooth muscle actin (α‐SMA) was used to assess pulmonary vasculature muscularization. A horizontal or vertical line was made across the shortest diameter of pulmonary vessels measuring 50‐200 μm. Thickness of the α‐SMA stained portion (along the line) was calculated over the external diameter of the vessel. All animal lungs in each group were evaluated by calculating α‐SMA thickness in five random vessels. Furthermore, positive staining for alpha‐SMA was quantified on all lungs using the Positive Pixel Count Algorithm (v9, Aperio Technologies, Inc., Vista, CA). Data are reported as positivity (number of positively stained pixels divided by the total number of pixels in the scanned image).

#### 2.6.5 Fibrosis

Masson's trichrome staining was done to evaluate collagen deposition in the perivascular, peribronchial, and alveoli. Ten fields were observed per lung at a magnification of ×20. ImageJ was used to determine percent staining in a section measuring 2181 μm × 1446 μm.

### 2.7 Right ventricular hypertrophy

A ratio of the thickness of the right ventricle to left ventricle+ septum on H&E stains of the heart was used as an index of right ventricular hypertrophy (RVH). To measure RVH, a series of seven horizontal lines spaced at 12 μm was placed over the heart images, and the length of the septum and ventricles were calculated.

### 2.8 qRT‐PCR

Total RNA was extracted from flash frozen tissue using TRIzol reagent and Phasemaker tube system (Thermo Fisher). Briefly, ~20 mg of tissue was homogenized in 1 mL of TRIzol using a beadbeater (BioSpec) for 60 seconds followed by 60 seconds rest on ice, then another 60 seconds of homogenization. The tissue homogenate was briefly spun to eliminate bubbles and transferred to the Phasemaker tubes. RNA extraction was then completed using the TRIzol manufacturer's instructions. Synthesis of cDNA was achieved using the Applied Biosystems High Capacity cDNA Reverse Transcription Kit (Thermo Fisher) with 2 μg of RNA. qRT‐PCR reactions were created using cDNA, EasyOligo custom primers (Sigma‐Aldrich, St. Louis, MO), and SYBR Green PCR Master Mix (Thermo Fisher). The BioRad CFX384 Touch Real‐Time PCR Detection System (BioRad, Hercules, CA) was used for PCR data acquisition. The program settings were as follows: 50°C for 2 minutes, 95°C for 10 minutes, 40 cycles of 95°C for 15 seconds, and 60°C for 1 minute, followed by a melt curve (0.5°C per cycle, 60 cycles stopping at 95°C). Starting quantity of RNA was calculated in CFX Manager Software (BioRad) using the relative standard curve method and data were normalized using β‐Actin as a housekeeping gene. Primers were designed using the protocol described in Thornton and Basu 2010 and are summarized in Supplementary Table S1.^33^ Primer selection was chosen before gene array analysis, but reflect genes known to be altered in BPD, including vasculogenesis, inflammation, fibrosis, cell proliferation, and apoptosis.

### 2.9 Gene array

Pathway‐specific gene expression was also analyzed using the RT2 Profiler PCR Array for Rat Endothelial Cell Biology (Qiagen, Hilden, Germany). In total, 96 genes from pathways associated with angiogenesis, vasoconstriction/vasodilation, inflammation, apoptosis, cell adhesion, coagulation, and platelet activation were profiled (for a complete listing of genes, see Supplementary Table S2). Clean‐up of RNA samples (n = all 24 animal lungs from the RA, BPD, and BPD + MSC group) with the RNeasy Mini Kit (Qiagen, Hilden, Germany) was performed prior to PCR profiling according to the manufacturer's instructions. Per the manufacturer's recommendation, on‐column DNA digestion was performed using the RNase‐Free DNase set (Qiagen, Hilden, Germany). RNA quality and concentration were determined using a spectrophotometer (BioTek Epoch, Winooski, VT) and cDNA was synthesized from 400 ng of total RNA using the RT2 First Strand Kit (Qiagen, Hilden, Germany) according to the manufacturer's protocol. RT2 Profiler reactions were then mixed, loaded into the plates, and run using the BioRad CFX384 PCR detection system according to the manufacturer's instructions. Data are reported as fold changes using the double‐delta CT formula.

### 2.10 Protein array

Lung lysate was analyzed using a rat cytokine array kit (Proteome Profiler, R&D Systems, Minneapolis, MN). Per manufacturer's directions, a total of 250 μg of tissue lysate was incubated in a nitrocellulose membrane array overnight at 4°C. Signals were visualized with chemiluminescence Western blot and developed on X‐ray film. The arrays were scanned, and optical densities were quantified using Image J software (NIH). The protein macroarray analysis included 79 cytokines/factors and included lung tissue protein samples from a subset of the three groups (n = all 24 animal lungs from the RA, BPD, and BPD + MSC groups).

## 3 STATISTICAL ANALYSIS

The data generated by the described experiments was evaluated using standard statistical approaches. Continuous data were expressed as mean ± SEM. Intergroup differences for parametric data were tested with one‐way ANOVA and Tukey's comparison test and nonparametric data with Kruskall Wallis followed by Dunn's test. Data were analyzed using STATA, (version 13, College Station, TX) and a *P* value <5% signifying statistical significance. Power analysis calculations were based on MLI differences between BPD and BPD + MSC groups in a published study demonstrating MSC efficacy in a study by Thebaud et al.^34^ Therefore, sample size to detect a difference, with a power of 80% and two‐sided α = .05, yielded four rats/group. We included at least eight animals per group to account for potential failure rates (ie, animal death prior to randomization into the four groups). Histologic and physiologic calculations were performed by study team members blinded to experimental groups.

## 4 RESULTS

### 4.1 Umbilical cord cells met minimum MSC criteria

Umbilical cord Wharton's jelly‐derived cells adhered to tissue culture dishes and displayed the characteristic cell surface antigens for MSC classification (Supplementary Figure S2). Cells were negative for hematologic stem cell markers HLA‐DR, CD117, and CD79 alpha. Consistent with the criteria set by International Society for Cell and Gene Therapy, cells differentiated into adipocytes, chondrocytes, and osteocytes when grown with lineage specific media, as previously describe.^20^

### 4.2 Survival rate and animal growth

A total of 35 animals were used in the experiments (RA n = 7, BPD n = 11, BPD + Veh n = 8, BPD + MSC n = 9). Survival rates among the groups were comparable (RA 100%, BPD 82%, and BPD + Veh = 100%, BPD + MSC 89%, log‐rank *P* = .6). Kaplan‐Meier curve is provided in Supplementary Figure S3.

On postnatal day 4 mean body weight of the rat pups was 10.0 ± 0.5 g. Weight of pups increased to approximately 20 g on day 10. Growth velocity did not differ between the groups (Supplementary Figure S4).

### 4.3 Nasal delivery of MSCs to the lungs

The pulmonary dispersion of human umbilical cord MSCs was monitored using immunohistochemistry of a human mitochondrial antibody. Supplementary Figure S5 provides evidence that intranasal administration of MSCs, at a dose of 5 × 10^5^, migrated towards the rat lungs.

### 4.4 Intranasal delivery of MSCs restore lung alveolarization

**Figure 2:**
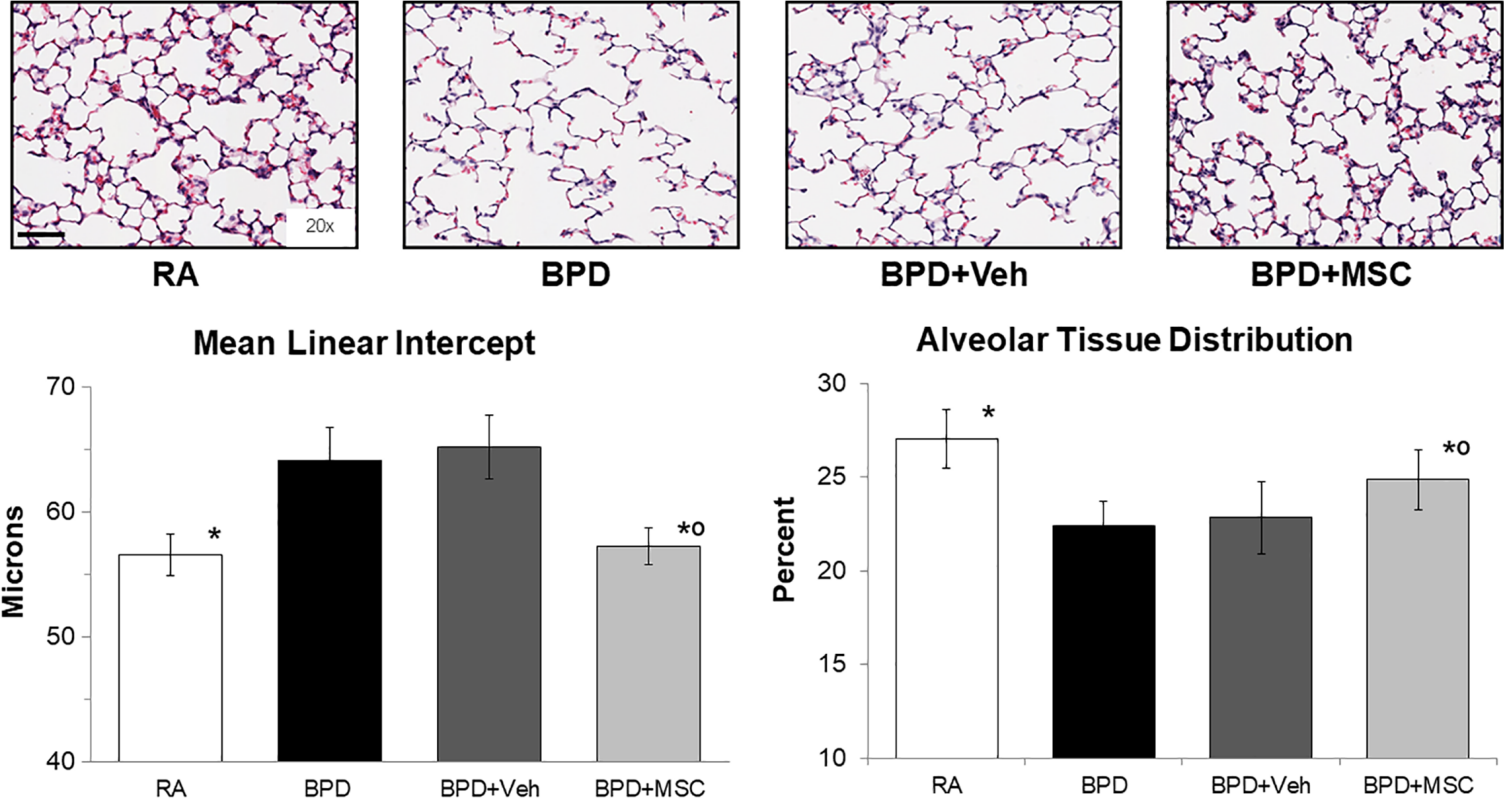
Intranasal delivery of mesenchymal stromal cells (MSCs) restores lung alveolarization. Hematoxylin and eosin stained lung sections show the characteristic simplification of lung alveolarization (larger, but fewer airspaces) in the hyperoxic injured bronchopulmonary dysplasia group (BPD). Using mean linear intercept as a surrogate for lung alveolar growth, intranasal delivery of MSCs fully restored lung architecture. Findings from alveolar tissue distribution corroborate histologic and mean linear intercept findings. Measurements expressed as mean ± SEM and included all 32 animals. RA, room air; Veh, vehicle. Mean linear intercepts: *P < .05 compared to BPD and BPD + Veh; °*P* > .05 compared to RA. Scale bar = 100 μm. Alveolar tissue distribution: *P < .05 compared to BPD and BPD + Veh, °*P* < .05 compared to RA

Exposure of hyperoxia created the characteristic histologic findings in human BPD. Figure 2 show representative light microscopic photographs of histological H&E stain differences between the groups. Specifically, animals that received 4 days of 60% O_2_ had fewer and larger sized alveoli, indicative of arrested alveolar development. Intranasal delivery of MSCs improved alveolarization, as measured by mean linear intercept (BPD 64.3 ± 2.6 μm vs BPD + MSC 57.3 ± 1.5 μm, *P* < .01) and alveolar tissue distribution. No difference was found comparing mean linear intercept in RA and BPD + MSC groups or BPD with BPD + Veh (*P* = .64 and .11, respectively).

### 4.5 Vascular development improved with MSCs given via the intranasal route

**Figure 3:**
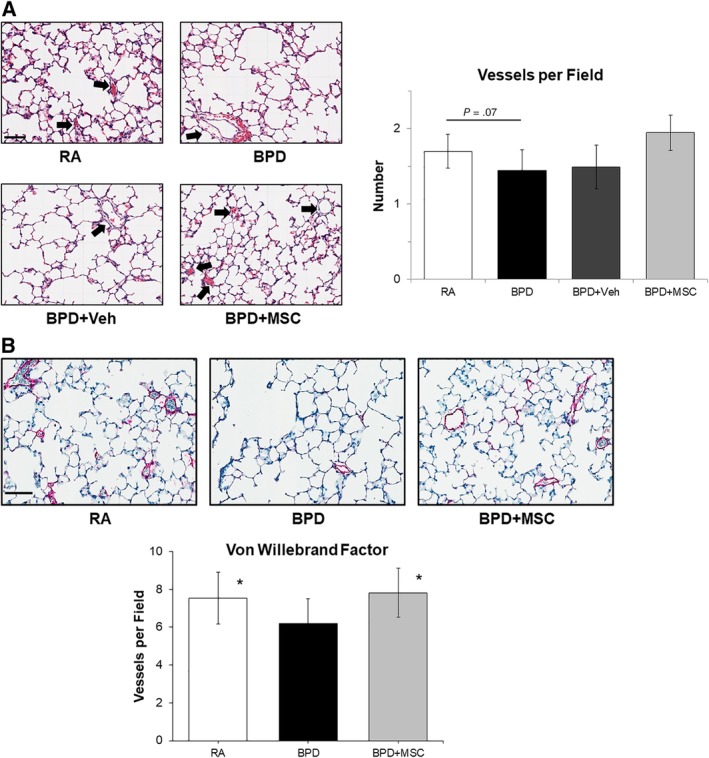
Intranasal delivery of mesenchymal stromal cells (MSCs) improves lung vascularization. A, Hematoxylin and eosin stained lung sections show a trend towards a decreased number of blood vessels in the hyperoxic injured bronchopulmonary dysplasia group (BPD). MSC therapy tended to have a higher blood vessel number. Measurements expressed as mean ± SEM and included all animals. Black arrows depict pulmonary vessels. Veh, vehicle. Scale bar = 100 μm, magnification at ×20. B, Von Willebrand Factor staining (magenta) of room air control animals (RA), BPD, and BPD + MSC depict pulmonary vascular development was restored after intranasal stem cell treatment. *P < .05 compared to BPD. Scale bar = 100 μm

To assess the effects of hyperoxic injury on vasculature development, the number of pulmonary vessels were counted on H&E stained lung sections (Figure 3A). Although significance was not obtained (*P* = .07), the pattern suggests that compared to RA rats, hyperoxic exposed animals trended towards a reduction in the number of blood vessels (1.4 ± 0.3 vs 1.7 ± 0.3 blood vessels/field, respectively, *P* = .07), whereas treatment with MSCs restored vessel number (1.9 ± 0.2 blood vessels/field).

To confirm the above findings, von Willebrand factor staining was conducted in the following cohorts: RA, BPD, and BPD + MSC animals. Similar to H&E results, hyperoxia‐induced BPD animals demonstrated a lower number of blood vessels when compared with RA animals (6.2 ± 1.3 vs 7.5 ± 1.4, *P* < .01; Figure 3B). Blood vessel number was restored in the BPD animals treated with MSCs (7.8 ± 1.3, *P* < .01compared to BPD; *P* = .5 compared to RA).

### 4.6 Smooth muscle thickening was attenuated with MSC and CdM therapy

**Figure 4:**
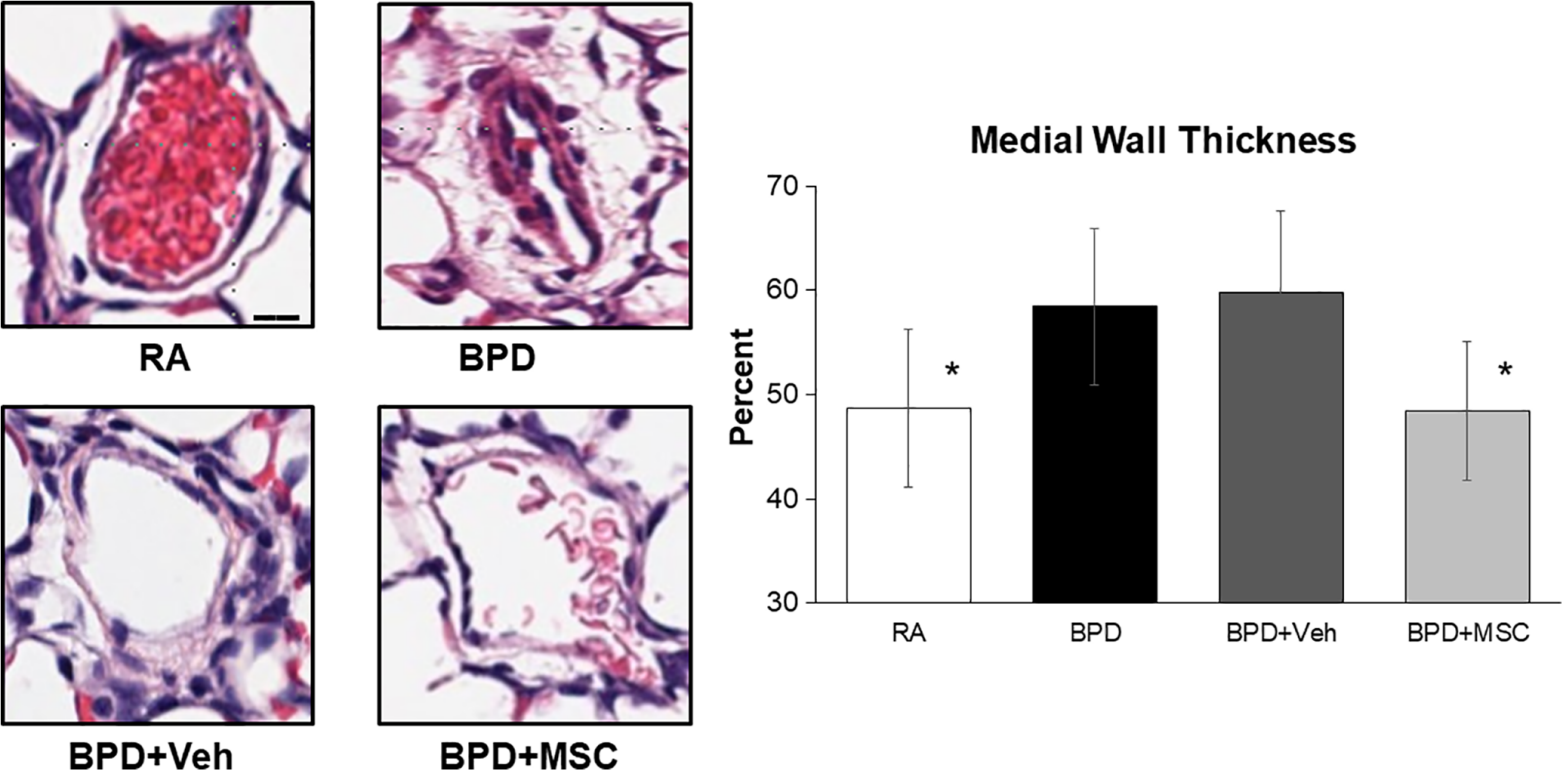
Intranasal delivery of mesenchymal stromal cells (MSCs) prevented early stages of pulmonary vascular remodeling in peripheral vessels. Hyperoxia‐exposed rats (BPD) had a significant increase in medial wall thickness in pulmonary vessels measuring <100 μm when compared to room air control animals (RA). Medial wall thickness in the BPD animals exposed to MSC therapy returned the levels to baseline. Measurements expressed as mean ± SEM in five random pulmonary vessels per lung in all animals. *P < .05 compared to BPD and BPD + Veh. Scale bar = 20 μm, magnification of hematoxylin and eosin images at ×80

To determine the effects of MSCs on pulmonary vascular remodeling, medial wall thickness (MWT) was determined in H&E lung sections. MWT was elevated in BPD and BPD + Veh animals (69.9% ± 7.1% and 64.3% ± 8.0%, respectively, Figure 4). MSC and CdM therapy significantly decreased MWT when compared to the BPD group.

### 4.7 Pulmonary vessel muscularization and cardiac remodeling was not elicited in our model

Vessel muscularization did not differ among the groups (Supplementary Figure S6) and cardiac RVH was not produced in any of the BPD groups (Supplementary Figure S6).

### 4.8 Lung fibrosis was similar among groups

Although collagen staining was seen in the peribronchial areas, it was not appreciable in the interstitium (data not shown).

### 4.9 VEGF mRNA lung expression increased after MSC treatment

MSC treatment increased vascular endothelial growth factor (VEGF) mRNA expression in lung homogenates compared to RA rats but did not differ from the BPD animals (Supplementary Figure S7). RNA levels of bax, caspase 3, interleukin‐6, interleukin‐10, timp 2, and transforming growth factor‐β were not affected by hyperoxia or MSC treatment.

### 4.10 Differential expression of genes between RA, BPD, and BPD + MSC animals

**Figure 5:**
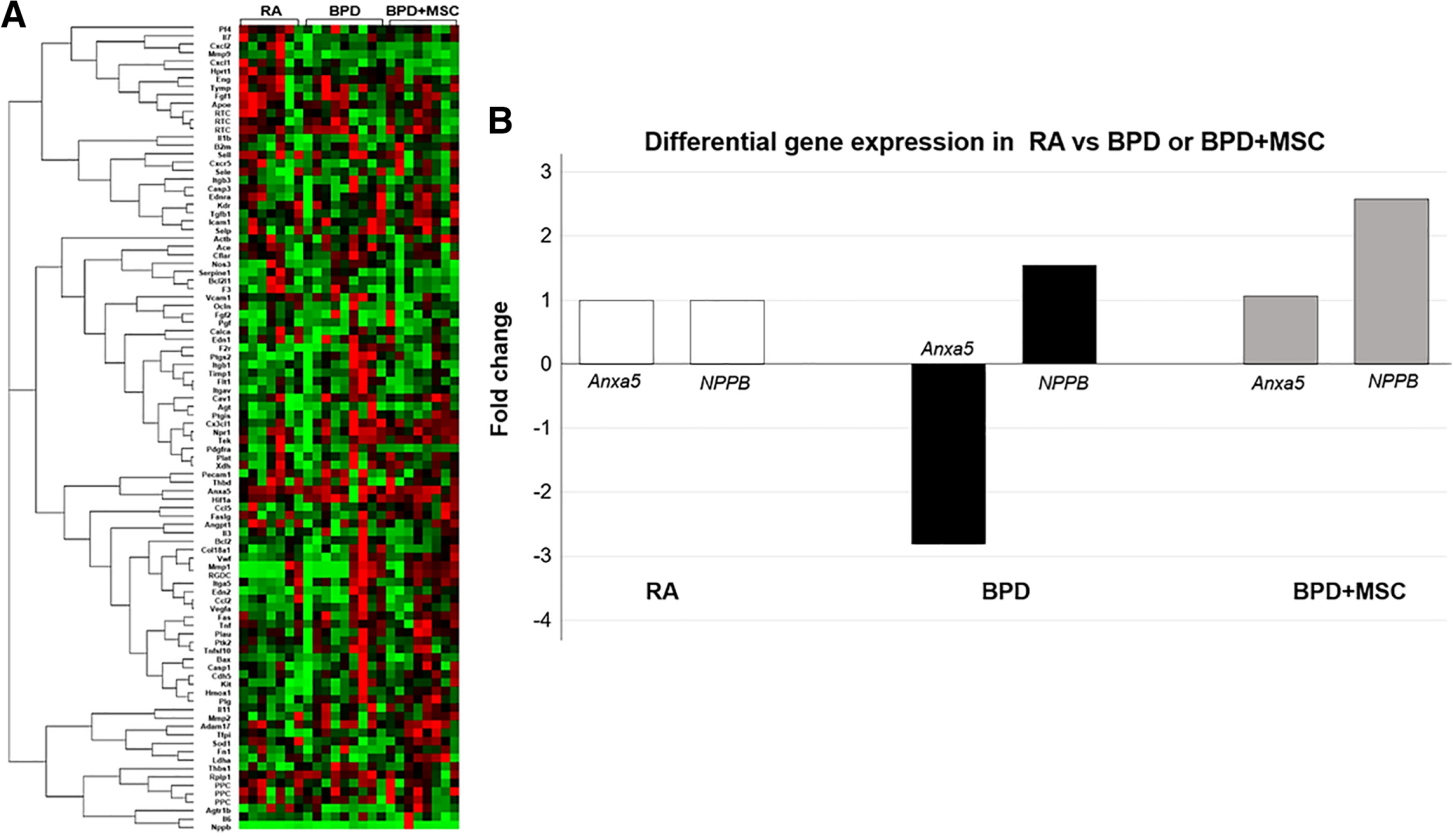
PCR gene array data demonstrates mesenchymal stromal cell (MSC) treatment in animals with hyperoxic lung injury influences biological processes, cellular components, and molecular function. A, A comparison analysis of gene expression was performed using the RT2 Profiler PCR Array for Rat Endothelial Cell Biology between the following animal groups: room air (RA) control, hyperoxia‐induced bronchopulmonary dysplasia (BPD), and BPD treated with MSCs (BPD + MSC). RNA was analyzed from rat lung tissue on day 20‐21 and included all animal lungs. Using a threshold of twofold expression change and statistical significance of *P* < .05, two separate genes were identified among the 3 groups. B, Genes differentially expressed in RA control animals compared to animals exposed to hyperoxia‐induced BPD and treated with MSCs

To identify the effect of MSCs on lung repair and restoration, differences in the mRNA levels of genes were examined using a PCR array and compared BPD + MSC to BPD and RA. We found that MSCs had an effect (≥twofold difference) in the expression profile of two genes: *Anxa5* (cell signaling, vesicle trafficking, cell division/migration, and apoptosis) and *NPPB* (cardiac remodeling, vasculogenesis, antimicrobial) (refer to Figure 5A).^35^, ^36^

### 4.11 MSCs express proteins that modulate inflammation, cell survival, and wound healing

**Figure 6:**
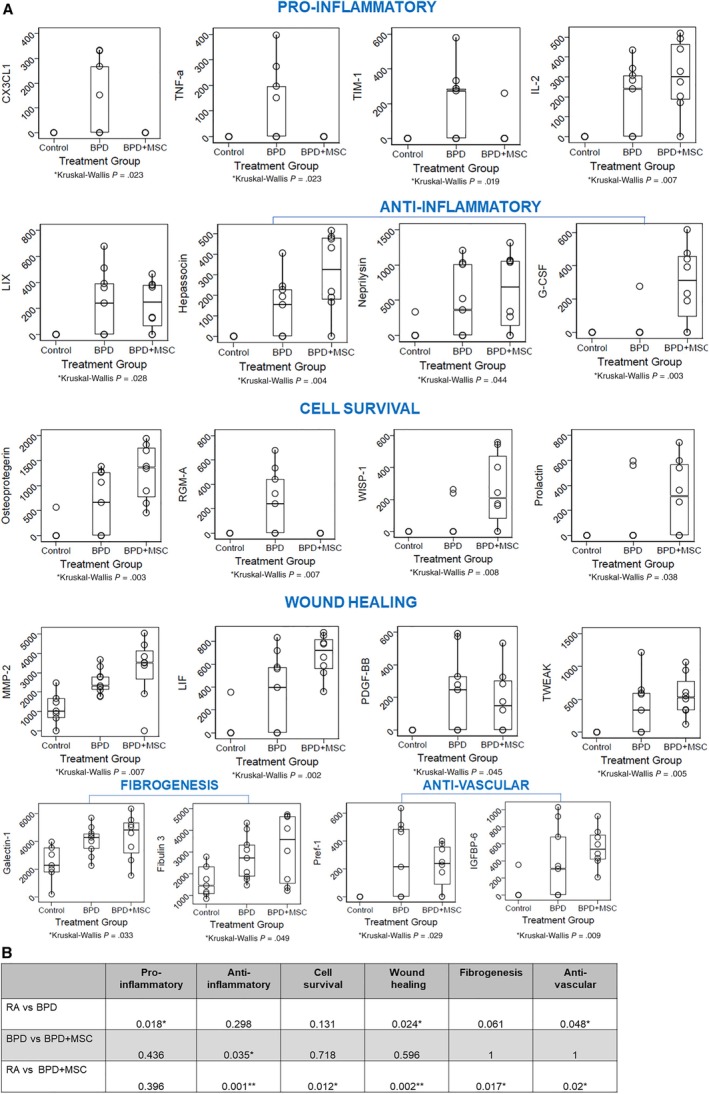
Protein analysis of lung homogenates from room air (RA) control, oxygen injured (bronchopulmonary dysplasia [BPD]), and BPD treated with mesenchymal stromal cells (BPD + MSCs). A, Select data displayed in the following grouping: Pro‐inflammatory; Anti‐inflammatory; Cell survival; Wound healing; Fibrogenesis; and Anti‐vascular. Data presented as mean ± SEM. N, all animals in each group. *P < .05 compared to BPD. CX3CL1, C‐X3‐C motif chemokine ligand 1; G‐CSF, granulocyte colony stimulating factor; IGFBP, insulin like growth factor binding protein; IL, interleukin; LIF, leukemia inhibitory factor; LIX, C‐X‐C motif chemokine 5; MMP, matrix metalloproteinase; PDGF‐BB, platelet derived growth factor receptor beta; Pref, preadipocyte factor; RGM‐A, repulsive guidance molecule BMP co‐receptor A; TIM, T‐cell immunoglobulin mucin receptor; TNF, tumor necrosis; TWEAK, TNF receptor superfamily member 12A; WISP, WNT1 inducible signaling pathway. B, Mean scores for the cytokines within their respective pathway. Of note, the RA group had multiple pathway differences compared to the other groups. However, only the anti‐inflammatory cytokines yielded significant between the BPD and BPMSC. The groups were examined using ANOVA, followed by pairwise comparisons using Bonferroni's adjusted significance level, where *P* < .05 was deemed significant

To investigate the effects of MSCs on lung protein expression we performed a protein macroarray. Of the 79 factors/cytokines tested, RA only expressed 30 proteins while BPD and BPD + MSC expressed 61 and 58 proteins, respectively. When compared to RA and BPD + MSC, BPD had four elevated proteins: CX3CL1, TNFα, TIM‐1, and RGM‐A. On the other hand, when compared to RA and BPD, MSCs showed an enriched expression of G‐CSF, WISP‐1, and prolactin. The lungs of MSC treated mice had significantly lower levels of BPD injury related to proteins associated with immunomodulation (CX3CL1, TNFα, TIM‐1, hepassocin, neprilysin), cell survival (osteoprotegerin), and wound healing (MMP‐2, LIF). Although proteins pertaining to fibrogenesis and anti‐vascular processes were different between RA and BPD + MSC groups, there was no significant difference between the BPD and BPD + MSC groups. The single protein that demonstrated a difference between the BPD and BPD + MSC group (post hoc analysis) was G‐CSF (*P* = .011). MSC treatment in this experimental model of BPD appears to impact inflammation when the genes were grouped into six processes (pro‐inflammation, anti‐inflammation, cell survival, wound healing, fibrogenesis, and anti‐vascular). Figure 6 summarizes the top proteins per functional category and sample protein membranes are found in Supplementary Figure S8. Supplementary Figure S8 also describes the statistical computation for the six processes and Supplementary Table S4 provides references for the groupings.

## 5 DISCUSSION

The major findings of the present study are that human umbilical cord tissue MSCs given: (a) intranasally is feasible and a noninvasive approach for delivery to the lungs, (b) can potentially restore lung alveolarization and vascularization in a moderate model of BPD, and (c) the restorative effects may, in part, be due to a concerted effort on angiogenesis, immunomodulation, wound healing, and cell survival.

The rationale for using MSCs as novel agents for BPD is based on their ability to secrete factors that contribute to tissue repair. MSCs are known to stimulate blood vessel formation, blunt inflammation, and promote alveolar epithelial cell survival and proliferation. While most preclinical BPD studies report the therapeutic action of these cells in BPD using an intratracheal and intravenous route, our project is the first to report intranasal delivery is a viable and efficacious approach (Supplementary Table S3). Previous reports by De Paepe et al attempted the intranasal route of stromal cells for BPD but found either no obvious pulmonary effect or worsening in lung development.^37^, ^38^ Conversely, a study in mice with ovalbumin‐induced asthma similarly demonstrated abrogation of airway injury after intranasal administration of MSCs.^39^ Additionally, these investigators demonstrated that intranasal delivery was more successful in reducing lung fibrosis, inflammation, and airway reactivity than the intravenous route.

Reasons that may explain the effectiveness in our study include: multiple dose administration, less severe BPD model, intranasal volume given, and anatomic positioning of animals during cell delivery.^40^, ^41^, ^42^ Moreover, using an automated cell counter we tested the viability of the human umbilical cord MSCs just prior to animal administration and our yield ranged between 93% and 99%.^20^ Despite being a crude approximation of cell viability, we wanted a quick and objective measure of the percent of live cells we were providing to the animals. Prior studies administering MSCs intranasally for lung disease do not mention stromal cell viability before animal delivery.

Intranasal administration of MSCs is a translationally relevant mode of administration that is noninvasive, rapidly absorbed, and offers a tremendous option for frequent dosing.^43^ Clinical advantages of the intranasal route include the ease of administration, whereby a patient does not require an intravenous line or endotracheal tube. Furthermore, intranasal delivery of products avoids the initial intestinal metabolism observed in intravenous, oral, or subcutaneous routes. The intranasal delivery of cells/drugs can also provide systemic effects, as the nasal mucosa is a highly vascularized tissue. In a study by Balyasnikova et al, intranasal delivery of labeled MSCs showed a high quantity of cells penetrating the lung parenchyma.^44^ These findings are opposite of those found by Reitz et al whereby neural stem/progenitor cells given intranasally to mice migrated to the brain and spleen but were not visualized in the lungs.^45^

Our study is in line with the literature showing that MSCs improve alveolarization and vascularization in animal models of BPD.^19^, ^34^, ^46^ Other investigators have established improved survival, animal growth, and exercise tolerance after MSC treatment.^19^, ^34^ We attribute the lack of these findings to the decreased severity of BPD in our animals. Our group chose to limit the injury model to the saccular stage (first four days in rodents) of lung development, as we believe this replicates the alveolar simplification and dysmorphic pulmonary vessel development observed in clinical practice (“new BPD”).^3^ This assumption is further strengthened by the fact that our model did not elicit late findings of pulmonary vascular remodeling (muscularization and RVH). These late findings of pulmonary vascular remodeling are observed in ~10% of neonates with severe BPD.^47^, ^48^ We also did not appreciate the lung fibrosis characteristic in other studies that induced BPD with higher oxygen levels and longer duration of the injury.^49^, ^50^, ^51^

Our gene data emphasizes the key roles angiogenesis and cell membrane repair mechanisms have on proper lung development. MSCs increased the expression of NPPB, a neovasculatory factor that stimulates endothelial regeneration.^36^, ^52^ The neovascular effects of NPPB are secondary to endothelial cell proliferation, migration, and recruitment of bone‐marrow derived endothelial progenitor cells.^36^, ^52^ Studies have clearly demonstrated the important role of the vascular endothelium during lung epithelial cell proliferation and maturation.^53^

A premature neonate is exposed to hyperoxia and ventilator‐associated trauma contributing to an impairment in the alveolar epithelial barrier. Normally, for cells to survive there must be a quick ability to reseal the membrane and restore epithelial barrier structure and function. However, this may be altered under chronic injury (eg, hyperoxia). For appropriate membrane repair, a series of steps must occur including an influx of calcium, activation of vesicular exocytosis, and upregulation of annexin production.^54^ Annexins act as environmental sensors that signal cell adaptation to stress, that are highly abundant in endothelial cells and have been implicated in in human airway epithelial cell membrane repair mechanisms.^35^, ^55^, ^56^, ^57^ This study demonstrated an increase in *anxa5* gene production in BPD animals treated with MSCs.

The immunomodulatory properties of MSCs make them novel and invaluable agents for the repair of lungs injured by chronic inflammation. Studies propose MSCs subdue immunologic responses by secreting soluble factors known to be immunosuppressive.^58^, ^59^, ^60^, ^61^ In our study, protein analysis of animals with hyperoxic lung injury treated with MSCs reduced the production of the pro‐inflammatory cytokines TNFα, CX3CL1, and TIM‐1. A growing number of translational studies support MSCs can regulate TNF signaling in BPD.^62^, ^63^ In a study examining the effects of human umbilical cord MSC microvesicles in renal injury, MSC microvesicles decreased inflammation through the downregulation of TNFα and CX3CL1.^64^ Additionally, the anti‐inflammatory roles of hepassocin and G‐CSF, found higher in our BPD + MSC cohort, have been the subject of recent studies.^65^, ^66^ Our stem cell treated animals had increased levels of lung neprilysin, a known factor that influences not only immune modulation but also pulmonary vascular growth.^67^ The upward trend in TGF‐β1 after MSC administration abrogates the activation of T cells and natural killer cells.^61^, ^63^ We were unable to replicate findings that have shown MSCs can increase the expression of IL‐6 (anti‐apoptotic) and IL‐10 (anti‐inflammatory) to reduce lung injury.^68^

Wound healing is a multifaceted process that requires a balanced response between re‐epithelialization, inflammation, and tissue granulation. After tissue injury, epithelial cells rapidly begin repairing the wound via secretion of MMPs and LIF.^69^, ^70^, ^71^, ^72^ During the inflammatory phase, platelets are recruited to curtail blood loss and macrophages and neutrophils migrate to the wound to remove debris. The extracellular matrix strengthens the initial clot and initiates scar formation.^73^ The final stage of healing focuses on remodeling the scar and regaining normal tissue function. These dynamic processes describes the tissue remodeling (Galectin‐1, Fibulin 3) seen in our study.^74^, ^75^ The protein microarray of rat lung homogenates with BPD also demonstrated cell survival properties after MSC delivery. For example, osteoprotegerin and WISP‐1 support cell survival in TNF‐induced apoptotic events and while prolactin is known for its role in reproductive functions it is also contains anti‐apoptotic properties.^76^, ^77^, ^78^, ^79^

Intranasal delivery of therapeutic agents may represent a less invasive strategy for treating critically ill neonates. Relatively few studies have investigated the efficacy of intranasal administration of therapeutic agents for lung disease, and to our knowledge none of the current literature has investigated the use of intranasally delivered human umbilical cord Wharton's jelly tissue MSCs for BPD. The current study suggests that intranasally delivered MSCs may represent a promising regenerative therapy for treating chronic lung disease in preterm neonates. However, as with all studies the current work should be interpreted with considerations for the limitations of the study. First, although the rodent model is well established and provides valuable insight into the pathophysiology of BPD, it is not a premature model and therefore does not represent the full milieu of illness experienced by preterm neonates. In retrospect, we also would have added a cohort of RA animals that received MSCs. In this way, we could have further demonstrated safety. Given the heightened production of numerous proteins following MSC therapy brings into question whether we needed to provide three intranasal doses. We cannot exclude that these results are a consequence of examining the proteomic expression 1 day after the last administration. Given the pleotropic effects of many of these cytokines another drawback is that the biologic processes we assigned may oversimplify their role/action in the body. Similar to intravenous and intratracheal routes, administering one dose of intranasal MSCs may suffice and may not imbalance or produce factors that when secreted chronically may potentially be harmful. In the future, our experiments will incorporate the efficacy of intranasal MSCs in a severe model of BPD and assess pulmonary function testing, fibrosis, and animal survival. Furthermore, we also plan to examine for MSC infiltration in other tissues including the brain, spleen, kidneys, and heart. These studies will be furthered by considering potential strategies to optimize or improve the proposed route of administration, such as: the timing and frequency of cell‐based or cell‐free therapies and the standardization of the regenerative preparation.

## 6 CONCLUSION

In summary, we provide evidence that the intranasal route of human umbilical cord tissue MSC delivery for BPD is effective in restoring lung alveolar growth and vascular development. Importantly, this study demonstrates that this noninvasive route of cell‐based administration may be given separately or as an adjunct/alternate to other routes. Our findings are translationally notable, given that premature neonates who develop BPD may no longer be intubated (but on noninvasive respiratory modes) nor require intravenous access (on full enteral volume). Ultimately, further studies will be necessary, however, this approach can theoretically be tested in other models of rodent lung injury.

## CONFLICT OF INTEREST

The authors indicated no potential conflicts of interest.

## AUTHOR CONTRIBUTIONS

A.M.: conception and design, financial support, collection and/or assembly of data, data analysis and interpretation, manuscript writing, final approval of the manuscript; C.W. and L.W.: conception and design, data analysis and interpretation, manuscript writing; J.J.: collection and/or assembly of data, final approval of the manuscript; M.J., M.N., K.Q., A.C., and T.S.: collection and/or assembly of data; M.P.: collection and/or assembly of data, illustrations; Y.A.: manuscript writing; S.M.: collection and/or assembly of data, data analysis and interpretation; P.H.: conception and design, manuscript writing, final approval of the manuscript; S.A.: data analysis and interpretation, final approval of the manuscript.

## Supporting information


**Supplementary Figure 1: Sample method used to calculate Alveolar Tissue Distribution.** Using MIPCloud hematoxylin and eosin lung sections were uploaded and standardized thresholds (200) and region of interest were obtained. The program computes the phasefraction (ie, alveolar tissue distribution). Scale bar = 100 μm.

**Supplementary Figure 2: Human umbilical cord cells met minimum mesenchymal stromal cell (MSC) criteria.** Umbilical cord cells had the characteristic fibroblast‐like morphology and adhered to plastic under standard culture conditions and expressed (magenta) specific cell surface antigen markers CD73, CD 90, and CD 105, while negative controls are seen in grey. Scale bar = 40 μm.

**Supplementary Figure 3: Kaplan‐Meier survival curve.** Survival outcomes between the 4 groups did not demonstrate statistical differences. RA = room air; BPD = bronchopulmonary dysplasia; Veh = vehicle, MSC = mesenchymal stromal cell.

**Supplementary Figure 4: Body weight curve showed no difference among groups.** RA = room air; BPD = bronchopulmonary dysplasia; Veh = vehicle, MSC = mesenchymal stromal cell.

**Supplementary Figure 5: Xenotransplantation of human umbilical cord MSCs via the nasal route migrated to the lungs in rats with hyperoxic injury.** Immunohistochemistry of rat lung sections stained for human mitochondrial antibody (brown, pointed by black arrows). Depicted are lung sections for 5 randomly chosen animals in the BPD + MSC cohort. Bars denote 50 μm.

**Supplementary Figure 6: Alpha smooth muscle actin (SMA) staining of pulmonary blood vessels and hematoxylin stained hearts.** No difference noted between the groups in pulmonary vessel muscularization nor right ventricle remodeling; n = all animals/group. RA = room air control; BPD = bronchopulmonary dysplasia; BPD + MSC = bronchopulmonary dysplasia treated with mesenchymal stomal cells. Scale bar for SMA = 10 μm and heart sections = 200 μm.

**Supplementary Figure 7: RT‐PCR data of rat lung homogenates.** IL‐interleukin, TIMP‐tissue inhibitors of metalloproteinases, TGF‐transforming growth factor, VEGF‐vascular endothelial growth factor. Data are shown as median with IQR. RA = room air control; BPD = bronchopulmonary dysplasia; BPD + MSC = bronchopulmonary dysplasia treated with mesenchymal stomal cells. N = all animals/group. * *P* < 0.05 compared to RA.

**Supplementary Figure 8: Sample protein array membranes for room air (RA) control, hyperoxia‐induced lung injury (BPD), and BPD animals treated with mesenchymal stromal cells (BPD + MSCs).** (A) blots (B) all plots (C) statistical methodology for processes. Labels below were taken from vendor (R&D) pamphlet.

**Supplementary Table 1 List of forward and reverse RNA primers. BAX = BCL2 associated x;** Casp = caspase; IL = interleukin; TIMP = tissue inhibitor of metalloproteinases; VEGF = vascular endothelial growth factor; TGF = transforming growth factor.

**Supplementary Table 2** List of genes tested in the RT2 Profiler PCR Array for Rat Endothelial Cell Biology.

**Supplementary Table 3** Advantages/disadvantages of the intranasal route for delivery of human umbilical cord mesenchymal stromal cells.

**Supplementary Table 4** References for groups of biologic processes.

## Data Availability

The data that support the findings of this study are available from the corresponding author upon reasonable request.

## References

[1] Johnson TJ , Patel AL , Jegier BJ , Engstrom JL , Meier PP . Cost of morbidities in very low birth weight infants. J Pediatr. 2013;162:243‐249.e1. 22910099 10.1016/j.jpeds.2012.07.013 PMC3584449

[2] Martin JA , Hamilton BE , Osterman MJK , et al. National Vital Statistics Reports Volume 64, Number 1, January 15, 2015.

[3] Jobe AH . The new bronchopulmonary dysplasia. Curr Opin Pediatr. 2011;23:167‐172. 21169836 10.1097/MOP.0b013e3283423e6b PMC3265791

[4] Digeronimo RJ , Mustafa SB , Ryan RM , et al. Mechanical ventilation down‐regulates surfactant protein a and keratinocyte growth factor expression in premature rabbits. Pediatr Res. 2007;62:277‐282. 17622950 10.1203/PDR.0b013e3181256aeb

[5] Doyle LW . Cardiopulmonary outcomes of extreme prematurity. Semin Perinatol. 2008;32:28‐34. 18249237 10.1053/j.semperi.2007.12.005

[6] Islam JY , Keller RL , Aschner JL , Hartert TV , Moore PE . Understanding the short‐ and long‐term respiratory outcomes of prematurity and bronchopulmonary dysplasia. Am J Respir Crit Care Med. 2015;192:134‐156. 26038806 10.1164/rccm.201412-2142PP PMC4532824

[7] Schmidt B , Asztalos EV , Roberts RS , et al. Impact of bronchopulmonary dysplasia, brain injury, and severe retinopathy on the outcome of extremely low‐birth‐weight infants at 18 months: results from the trial of indomethacin prophylaxis in preterms. JAMA. 2003;289:1124‐1129. 12622582 10.1001/jama.289.9.1124

[8] Collins JJP , Tibboel D , de Kleer IM , et al. The future of bronchopulmonary dysplasia: emerging pathophysiological concepts and potential new avenues of treatment. Front Med. 2017;4:61. 10.3389/fmed.2017.00061 PMC5439211 28589122

[9] Coalson JJ . Pathology of new bronchopulmonary dysplasia. Semin Neonatol. 2003;8:73‐81. 12667832 10.1016/s1084-2756(02)00193-8

[10] Chess PR , D'Angio CT , Pryhuber GS , Maniscalco WM . Pathogenesis of bronchopulmonary dysplasia. Semin Perinatol. 2006;30:171‐178. 16860156 10.1053/j.semperi.2006.05.003

[11] Mason C , Dunnill P . A brief definition of regenerative medicine. Regen Med. 2008;3:1‐5. 18154457 10.2217/17460751.3.1.1

[12] Hunziker R . Regenerative medicine—fact sheet. Natl Inst Health. 2010;1‐2.

[13] Gupta N , Su X , Popov B , Lee JW , Serikov V , Matthay MA . Intrapulmonary delivery of bone marrow‐derived mesenchymal stem cells improves survival and attenuates endotoxin‐induced acute lung injury in mice. J Immunol. 2007;179:1855‐1863. 17641052 10.4049/jimmunol.179.3.1855

[14] Fang X , Neyrinck AP , Matthay MA , Lee JW . Allogeneic human mesenchymal stem cells restore epithelial protein permeability in cultured human alveolar type II cells by secretion of angiopoietin‐1. J Biol Chem. 2010;285:26211‐26222. 20554518 10.1074/jbc.M110.119917 PMC2924032

[15] Bernardo ME , Pagliara D , Locatelli F . Mesenchymal stromal cell therapy: a revolution in regenerative medicine? Bone Marrow Transplant. 2012;47:164‐171. 21478914 10.1038/bmt.2011.81

[16] Chang YS , Choi SJ , Sung DK , et al. Intratracheal transplantation of human umbilical cord blood derived mesenchymal stem cells dose‐dependently attenuates hyperoxia‐induced lung injury in neonatal rats. Cell Transplant. 2011;12:1‐32. 10.3727/096368911X565038 23167961

[17] Baber SR , Deng W , Master RG , et al. Intratracheal mesenchymal stem cell administration attenuates monocrotaline‐induced pulmonary hypertension and endothelial dysfunction. Am J Physiol Heart Circ Physiol. 2007;292:H1120‐H1128. 16980338 10.1152/ajpheart.00173.2006

[18] Sung DK , Chang YS , Ahn SY , et al. Optimal route for human umbilical cord blood‐derived mesenchymal stem cell transplantation to protect against neonatal hyperoxic lung injury: gene expression profiles and histopathology. PLoS One. 2015;10:e0135574. 26305093 10.1371/journal.pone.0135574 PMC4549285

[19] Pierro M , Ionescu L , Montemurro T , et al. Short‐term, long‐term and paracrine effect of human umbilical cord‐derived stem cells in lung injury prevention and repair in experimental bronchopulmonary dysplasia. Thorax. 2013;68:475‐484. 23212278 10.1136/thoraxjnl-2012-202323

[20] Balgi‐Agarwal S , Winter C , Corral A , Mustafa SB , Hornsby P , Moreira A . Comparison of preterm and term Wharton's jelly‐derived mesenchymal stem cell properties in different oxygen tensions. Cells Tissues Organs. 2018;205:137‐150. 29949803 10.1159/000489256 PMC6117836

[21] Augustine S , Avey MT , Harrison B , et al. Mesenchymal stromal cell therapy in bronchopulmonary dysplasia: systematic review and meta‐analysis of preclinical studies. Stem Cells Translational Medicine. 2017;6:2079‐2093. 29045045 10.1002/sctm.17-0126 PMC5702524

[22] Leary S , Das P , Ponnalagu D , et al. Genetic strain and sex differences in a hyperoxia‐induced mouse model of varying severity of bronchopulmonary dysplasia. Am J Pathol. 2019;189:999‐1014. 30794808 10.1016/j.ajpath.2019.01.014 PMC6526502

[23] Balany J , Bhandari V . Understanding the impact of infection, inflammation, and their persistence in the pathogenesis of bronchopulmonary dysplasia. Front Med. 2015;2:90. 10.3389/fmed.2015.00090 PMC4685088 26734611

[24] Yee M , Chess PR , McGrath‐Morrow SA , et al. Neonatal oxygen adversely affects lung function in adult mice without altering surfactant composition or activity. Am J Physiol Cell Mol Physiol. 2009;297:L641‐L649. 10.1152/ajplung.00023.2009 PMC2770788 19617311

[25] Berger J , Bhandari V . Animal models of bronchopulmonary dysplasia. The term mouse models. Am J Physiol Cell Mol Physiol. 2014;307:L936‐L947. 10.1152/ajplung.00159.2014 PMC4269689 25305249

[26] Stewart K , Schroeder VA . Rodent identification II. J Vis Exp. 2017; http://jove.com/science-education/10189/rodent-identification-i.

[27] Hanson LR , Fine JM , Svitak AL , Faltesek KA . Intranasal administration of CNS therapeutics to awake mice. J Vis Exp. 2013; http://jove.com/video/4440/intranasal-administration-of-cns-therapeutics-to-awake-mice. 10.3791/4440 PMC3653240 23608783

[28] Danielyan L , Schäfer R , von Ameln‐Mayerhofer A , et al. Intranasal delivery of cells to the brain. Eur J Cell Biol. 2009;88:315‐324. 19324456 10.1016/j.ejcb.2009.02.001

[29] Galeano C , Qiu Z , Mishra A , et al. The route by which intranasally delivered stem cells enter the central nervous system. Cell Transplant. 2018;27:501‐514. 29756518 10.1177/0963689718754561 PMC6038044

[30] Allard J , Li K , Lopez XM , et al. Immunohistochemical toolkit for tracking and quantifying xenotransplanted human stem cells. Regen Med. 2014;9:437‐452. 25159062 10.2217/rme.14.26 PMC4161450

[31] Jacob RE , Carson JP , Gideon KM , Amidan BG , Smith CL , Lee KM . Comparison of two quantitative methods of discerning airspace enlargement in smoke‐exposed mice. PLoS One. 2009;4:e6670. 19688093 10.1371/journal.pone.0006670 PMC2722737

[32] Kim KC , Lee HR , Kim SJ , Cho MS , Hong YM . Changes of gene expression after bone marrow cell transfusion in rats with monocrotaline‐induced pulmonary hypertension. J Korean Med Sci. 2012;27:605‐613. 22690090 10.3346/jkms.2012.27.6.605 PMC3369445

[33] Thornton B , Basu C . Real‐time PCR (qPCR) primer design using free online software. Biochem Mol Biol Educ. 2011;39:145‐154. 21445907 10.1002/bmb.20461

[34] van Haaften T , Byrne R , Bonnet S , et al. Airway delivery of mesenchymal stem cells prevents arrested alveolar growth in neonatal lung injury in rats. Am J Respir Crit Care Med. 2009;180:1131‐1142. 19713449 10.1164/rccm.200902-0179OC PMC3269236

[35] Monastyrskaya K , Babiychuk EB , Draeger A . The annexins: spatial and temporal coordination of signaling events during cellular stress. Cell Mol Life Sci. 2009;66:2623‐2642. 19381436 10.1007/s00018-009-0027-1 PMC11115530

[36] Shmilovich H , Ben‐Shoshan J , Tal R , et al. B‐type natriuretic peptide enhances vasculogenesis by promoting number and functional properties of early endothelial progenitor cells. Tissue Eng Part A. 2009;15:2741‐2749. 19275472 10.1089/ten.TEA.2008.0414

[37] Liu L , Mao Q , Chu S , et al. Intranasal versus intraperitoneal delivery of human umbilical cord tissue‐derived cultured mesenchymal stromal cells in a murine model of neonatal lung injury. Am J Pathol. 2014;184:3344‐3358. 25455688 10.1016/j.ajpath.2014.08.010

[38] Fritzell JA , Mao Q , Gundavarapu S , et al. Fate and effects of adult bone marrow cells in lungs of normoxic and hyperoxic newborn mice. Am J Respir Cell Mol Biol. 2009;40:575‐587. 18988921 10.1165/rcmb.2008-0176OC PMC2677437

[39] Royce SG , Rele S , Broughton BRS , Kelly K , Samuel CS . Intranasal administration of mesenchymoangioblast‐derived mesenchymal stem cells abrogates airway fibrosis and airway hyperresponsiveness associated with chronic allergic airways disease. FASEB J. 2017;31:4168‐4178. 28626025 10.1096/fj.201700178R

[40] Southam DS , Dolovich M , O'Byrne PM , et al. Distribution of intranasal instillations in mice: effects of volume, time, body position, and anesthesia. Am J Physiol Cell Mol Physiol. 2002;282:L833‐L839. 10.1152/ajplung.00173.2001 11880310

[41] Dao DT , Vuong JT , Anez‐Bustillos L , et al. Intranasal delivery of VEGF enhances compensatory lung growth in mice. PLoS One. 2018;13:e0198700. 29879188 10.1371/journal.pone.0198700 PMC5991715

[42] Miller MA , Stabenow JM , Parvathareddy J , et al. Visualization of murine intranasal dosing efficiency using luminescent *Francisella tularensis*: effect of instillation volume and form of Anesthesia. PLoS One. 2012;7:e31359. 22384012 10.1371/journal.pone.0031359 PMC3286442

[43] Grassin‐Delyle S , Buenestado A , Naline E , et al. Intranasal drug delivery: an efficient and non‐invasive route for systemic administration. Pharmacol Ther. 2012;134:366‐379. 22465159 10.1016/j.pharmthera.2012.03.003

[44] Balyasnikova IV , Prasol MS , Ferguson SD , et al. Intranasal delivery of mesenchymal stem cells significantly extends survival of irradiated mice with experimental brain tumors. Mol Ther. 2014;22:140‐148. 24002694 10.1038/mt.2013.199 PMC3978787

[45] Reitz M , Demestre M , Sedlacik J , et al. Intranasal delivery of neural stem/progenitor cells: a noninvasive passage to target intracerebral glioma. Stem Cells Translational Medicine. 2012;1:866‐873. 23283548 10.5966/sctm.2012-0045 PMC3659670

[46] Chang YS , Oh W , Choi SJ , et al. Human umbilical cord blood‐derived mesenchymal stem cells attenuate hyperoxia‐induced lung injury in neonatal rats. Cell Transplant. 2009;18:869‐886. 19500472 10.3727/096368909X471189

[47] Abman SH . Pulmonary vascular disease and bronchopulmonary dysplasia: evaluation and treatment of pulmonary hypertension. Neoreviews. 2011;12:e645‐e651.

[48] Steinhorn RH . Neonatal pulmonary hypertension. Pediatr Crit Care Med. 2010;11:S79‐S84. 20216169 10.1097/PCC.0b013e3181c76cdc PMC2843001

[49] D'Angio CT , Ryan RM . Animal models of bronchopulmonary dysplasia. The preterm and term rabbit models. Am J Physiol Cell Mol Physiol. 2014;307:L959‐L969. 10.1152/ajplung.00228.2014 25326582

[50] Bui CB , Pang MA , Sehgal A , et al. Pulmonary hypertension associated with bronchopulmonary dysplasia in preterm infants. J Reprod Immunol. 2017;124:21‐29. 29035757 10.1016/j.jri.2017.09.013

[51] Royce SG , Nold MF , Bui C , et al. Airway remodeling and hyperreactivity in a model of bronchopulmonary dysplasia and their modulation by IL‐1 receptor antagonist. Am J Respir Cell Mol Biol. 2016;55:858‐868. 27482635 10.1165/rcmb.2016-0031OC

[52] Kuhn M . Endothelial actions of atrial and B‐type natriuretic peptides. Br J Pharmacol. 2012;166:522‐531. 22220582 10.1111/j.1476-5381.2012.01827.x PMC3417485

[53] Yao J , Guihard PJ , Wu X , et al. Vascular endothelium plays a key role in directing pulmonary epithelial cell differentiation. J Cell Biol. 2017;216:3369‐3385. 28838957 10.1083/jcb.201612122 PMC5626536

[54] Cooper ST , McNeil PL . Membrane repair: mechanisms and pathophysiology. Physiol Rev. 2015;95:1205‐1240. 26336031 10.1152/physrev.00037.2014 PMC4600952

[55] Monastyrskaya K . Functional association between regulatory RNAs and the annexins. Int J Mol Sci. 2018;19:591. 10.3390/ijms19020591 PMC5855813 29462943

[56] Bouter A , Gounou C , Bérat R , et al. Annexin‐A5 assembled into two‐dimensional arrays promotes cell membrane repair. Nat Commun. 2011;2:270. 21468022 10.1038/ncomms1270 PMC3104517

[57] Patchell BJ , Wojcik KR , Yang T‐L , White SR , Dorscheid DR . Glycosylation and annexin II cell surface translocation mediate airway epithelial wound repair. Am J Physiol Cell Mol Physiol. 2007;293:L354‐L363. 10.1152/ajplung.00412.2006 17513451

[58] Shi M , Liu ZW , Wang FS . Immunomodulatory properties and therapeutic application of mesenchymal stem cells. Clin Exp Immunol. 2011;164:1‐8. 10.1111/j.1365-2249.2011.04327.x PMC3074211 21352202

[59] Gao F , Chiu SM , Motan DAL , et al. Mesenchymal stem cells and immunomodulation: current status and future prospects. Cell Death Dis. 2016;7:e2062. 26794657 10.1038/cddis.2015.327 PMC4816164

[60] Boshuizen MCS , Steinberg GK . Stem cell‐based immunomodulation after stroke: effects on brain repair processes. Stroke. 2018;49:1563‐1570. 29724892 10.1161/STROKEAHA.117.020465 PMC6063361

[61] Nauta AJ , Fibbe WE . Immunomodulatory properties of mesenchymal stromal cells. Blood. 2007;110:3499‐3506. 17664353 10.1182/blood-2007-02-069716

[62] Chang YS , Choi SJ , Ahn SY , et al. Timing of umbilical cord blood derived mesenchymal stem cells transplantation determines therapeutic efficacy in the neonatal hyperoxic lung injury. PLoS One. 2013;8:e52419. 23349686 10.1371/journal.pone.0052419 PMC3549907

[63] Monsel A , Zhu Y , Gennai S , Hao Q , Liu J , Lee JW . Cell‐based therapy for acute organ injury. Anesthesiology. 2014;121:1099‐1121. 25211170 10.1097/ALN.0000000000000446 PMC4206665

[64] Zou X , Zhang G , Cheng Z , et al. Microvesicles derived from human Wharton's jelly mesenchymal stromal cells ameliorate renal ischemia‐reperfusion injury in rats by suppressing CX3CL1. Stem Cell Res Ther. 2014;5:40. 24646750 10.1186/scrt428 PMC4055103

[65] Wang J , Sanmamed MF , Datar I , et al. Fibrinogen‐like protein 1 is a major immune inhibitory ligand of LAG‐3. Cell. 2019;176:334‐347.e12. 30580966 10.1016/j.cell.2018.11.010 PMC6365968

[66] Jeon GW , Sung DK , Jung YJ , et al. Granulocyte colony stimulating factor attenuates hyperoxia‐induced lung injury by down‐modulating inflammatory responses in neonatal rats. Yonsei Med J. 2011;52:65‐73. 21155037 10.3349/ymj.2011.52.1.65 PMC3017710

[67] Dempsey EC , Wick MJ , Karoor V , et al. Neprilysin null mice develop exaggerated pulmonary vascular remodeling in response to chronic hypoxia. Am J Pathol. 2009;174:782‐796. 19234135 10.2353/ajpath.2009.080345 PMC2665740

[68] Walter J , Ware LB , Matthay MA . Mesenchymal stem cells: mechanisms of potential therapeutic benefit in ARDS and sepsis. Lancet Respir Med. 2014;2:1016‐1026. 25465643 10.1016/S2213-2600(14)70217-6

[69] Caley MP , Martins VLC , O'Toole EA . Metalloproteinases and wound healing. Adv Wound Care. 2015;4:225‐234. 10.1089/wound.2014.0581 PMC4397992 25945285

[70] Akita S , Daian T , Ishihara H , Fujii T , Akino K . Leukemia inhibitory factor‐transfected embryonic fibroblasts and vascular endothelial growth factor successfully improve the skin substitute wound healing by increasing angiogenesis and matrix production. J Dermatol Sci. 2004;36:11‐23. 15488701 10.1016/j.jdermsci.2004.05.007

[71] Foronjy RF , Dabo AJ , Cummins N , Geraghty P . Leukemia inhibitory factor protects the lung during respiratory syncytial viral infection. BMC Immunol. 2014;15:41. 25277705 10.1186/s12865-014-0041-4 PMC4189665

[72] Chromy BA , Eldridge A , Forsberg JA , et al. Wound outcome in combat injuries is associated with a unique set of protein biomarkers. J Transl Med. 2013;11:281. 24192341 10.1186/1479-5876-11-281 PMC3827499

[73] Olczyk P , Mencner Ł , Komosinska‐Vassev K . The role of the extracellular matrix components in cutaneous wound healing. Biomed Res Int. 2014;2014:1‐8. 10.1155/2014/747584 PMC3977088 24772435

[74] Kathiriya JJ , Nakra N , Nixon J , et al. Galectin‐1 inhibition attenuates profibrotic signaling in hypoxia‐induced pulmonary fibrosis. Cell Death Discov. 2017;3:17010. 28417017 10.1038/cddiscovery.2017.10 PMC5385413

[75] Mabotuwana N , Murtha L , Hardy S , Boyle A . Fibulin‐3 in cardiac fibrosis. Hear Lung Circ. 2017;26:S133.

[76] Venkatesan B , Prabhu SD , Venkatachalam K , et al. WNT1‐inducible signaling pathway protein‐1 activates diverse cell survival pathways and blocks doxorubicin‐induced cardiomyocyte death. Cell Signal. 2010;22:809‐820. 20074638 10.1016/j.cellsig.2010.01.005 PMC2885703

[77] Venkatachalam K , Venkatesan B , Valente AJ , et al. WISP1, a pro‐mitogenic, pro‐survival factor, mediates tumor necrosis factor‐α (TNF‐α)‐stimulated cardiac fibroblast proliferation but inhibits TNF‐α‐induced cardiomyocyte death. J Biol Chem. 2009;284:14414‐14427. 19339243 10.1074/jbc.M809757200 PMC2682890

[78] Pritzker LB , Scatena M , Giachelli CM . The role of osteoprotegerin and tumor necrosis factor‐related apoptosis‐inducing ligand in human microvascular endothelial cell survival. Mol Biol Cell. 2004;15:2834‐2841. 15064358 10.1091/mbc.E04-01-0059 PMC420106

[79] Ben‐Jonathan N , LaPensee CR , LaPensee EW . What can we learn from rodents about prolactin in humans? Endocr Rev. 2008;29:1‐41. 18057139 10.1210/er.2007-0017 PMC2244934

