# Supplementary figures and images for: Intranasal delivery of human umbilical cord Wharton's jelly mesenchymal stromal cells restores lung alveolarization and vascularization in experimental bronchopulmonary dysplasia
Source: Stem Cells Transl Med. 2019 Nov 27;9(2):221–34. doi: 10.1002/sctm.18-0273 (PMC6988765; doi:10.1002/sctm.18-0273)

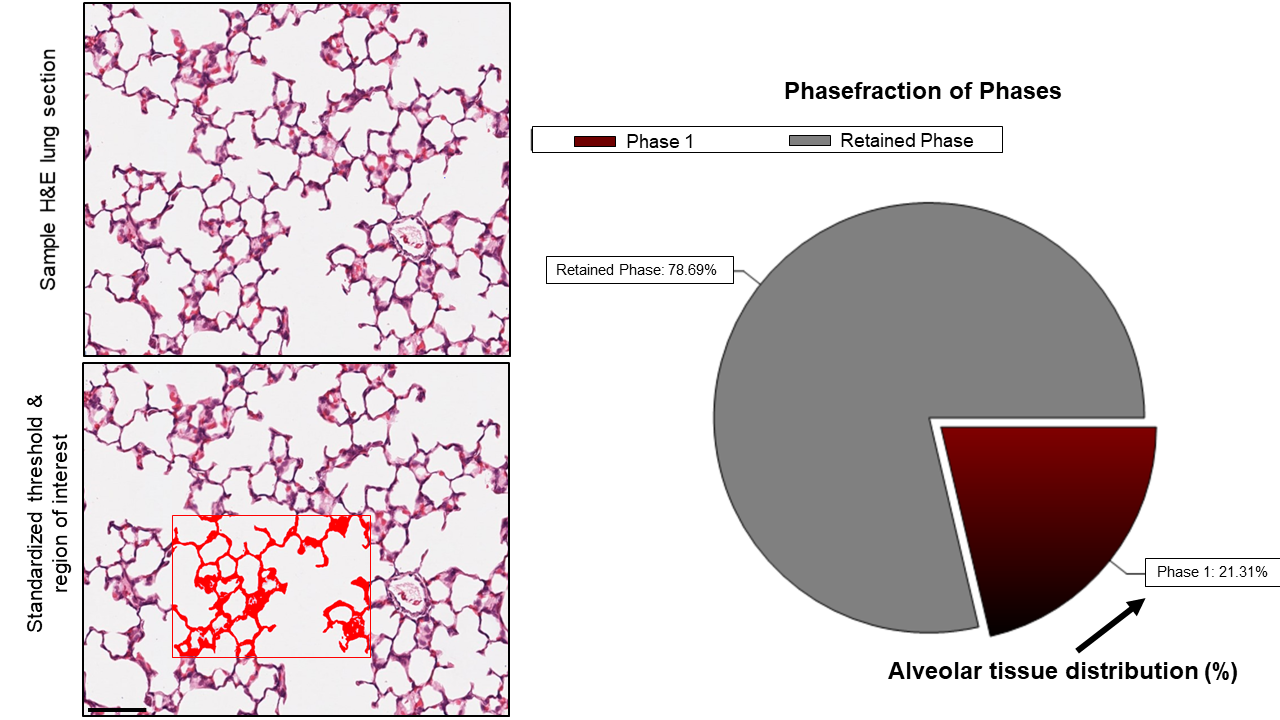

Supplement: Supplementary file 1 — Supplementary Figure 1: Sample method used to calculate Alveolar Tissue Distribution. Using MIPCloud hematoxylin and eosin lung sections were uploaded and standardized thresholds (200) and region of interest were obtained. The program computes the phasefraction (ie, alveolar tissue distribution). Scale bar = 100 μm. [file SCT3-9-221-s001.tif]

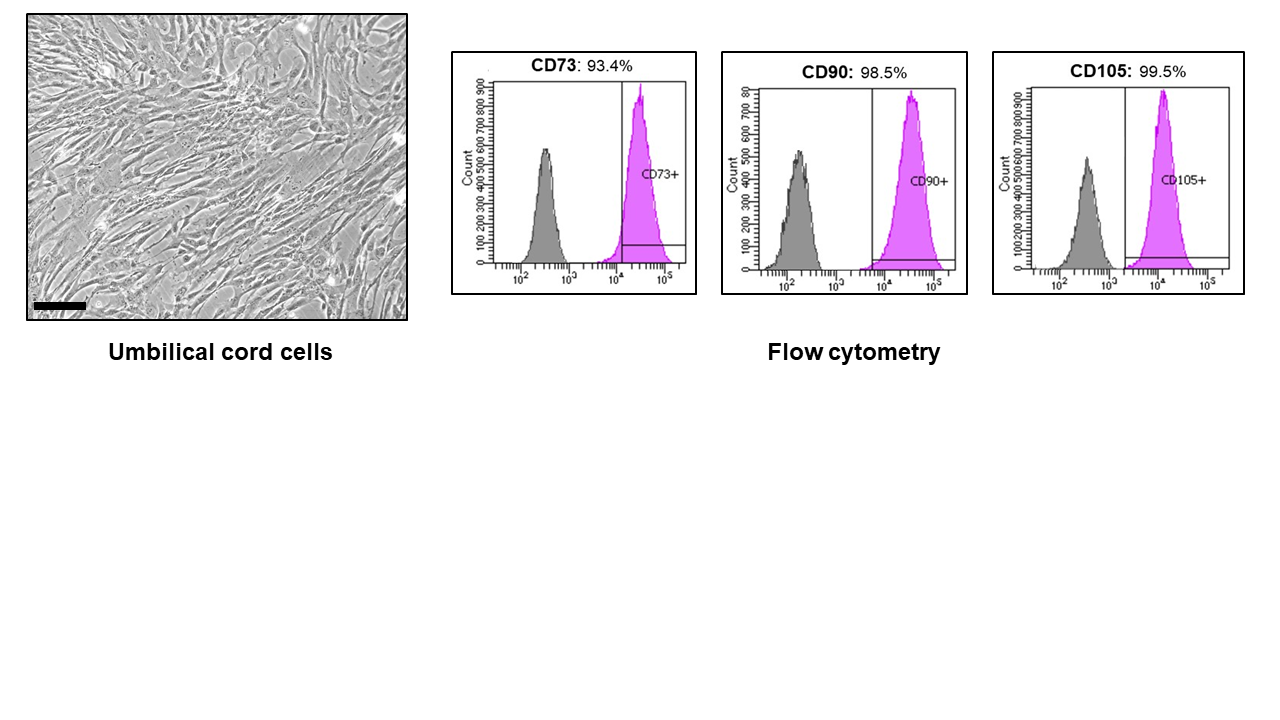

Supplement: Supplementary file 2 — Supplementary Figure 2: Human umbilical cord cells met minimum mesenchymal stromal cell (MSC) criteria. Umbilical cord cells had the characteristic fibroblast‐like morphology and adhered to plastic under standard culture conditions and expressed (magenta) specific cell surface antigen markers CD73, CD 90, and CD 105, while negative controls are seen in grey. Scale bar = 40 μm. [file SCT3-9-221-s002.tif]

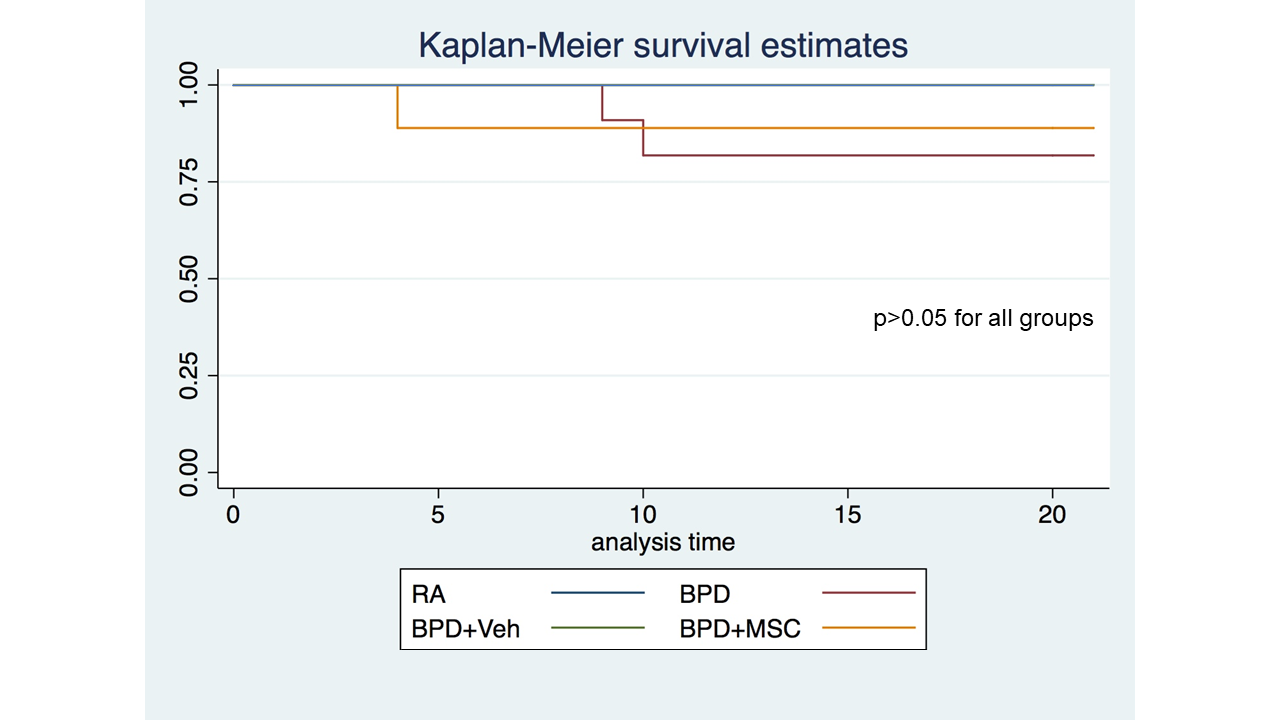

Supplement: Supplementary file 3 — Supplementary Figure 3: Kaplan‐Meier survival curve. Survival outcomes between the 4 groups did not demonstrate statistical differences. RA = room air; BPD = bronchopulmonary dysplasia; Veh = vehicle, MSC = mesenchymal stromal cell. [file SCT3-9-221-s003.tif]

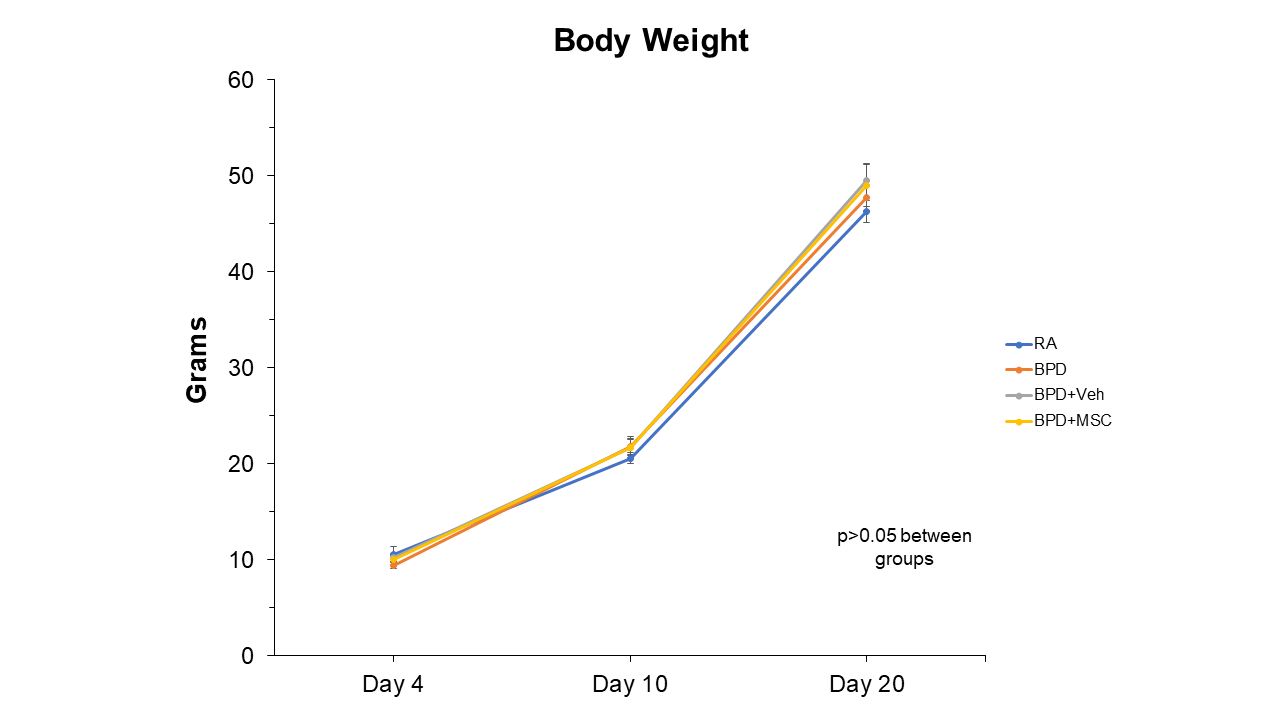

Supplement: Supplementary file 4 — Supplementary Figure 4: Body weight curve showed no difference among groups. RA = room air; BPD = bronchopulmonary dysplasia; Veh = vehicle, MSC = mesenchymal stromal cell. [file SCT3-9-221-s004.tif]

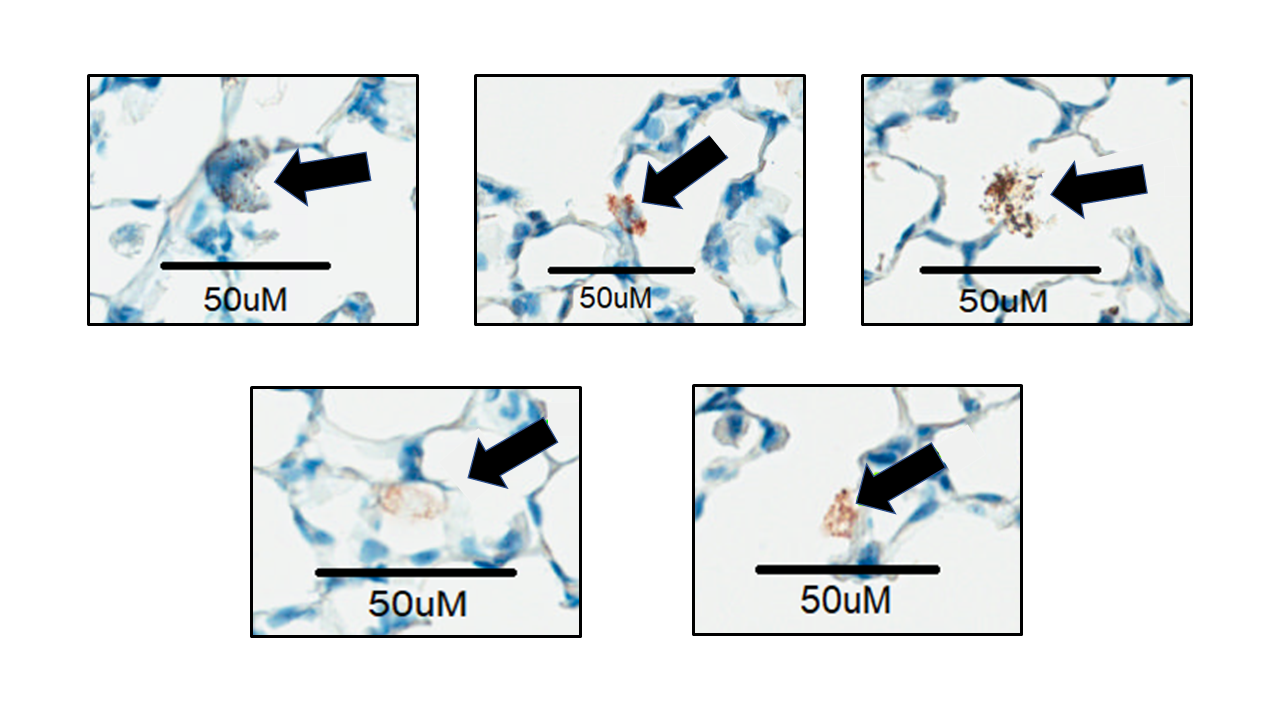

Supplement: Supplementary file 5 — Supplementary Figure 5: Xenotransplantation of human umbilical cord MSCs via the nasal route migrated to the lungs in rats with hyperoxic injury. Immunohistochemistry of rat lung sections stained for human mitochondrial antibody (brown, pointed by black arrows). Depicted are lung sections for 5 randomly chosen animals in the BPD + MSC cohort. Bars denote 50 μm. [file SCT3-9-221-s005.tif]

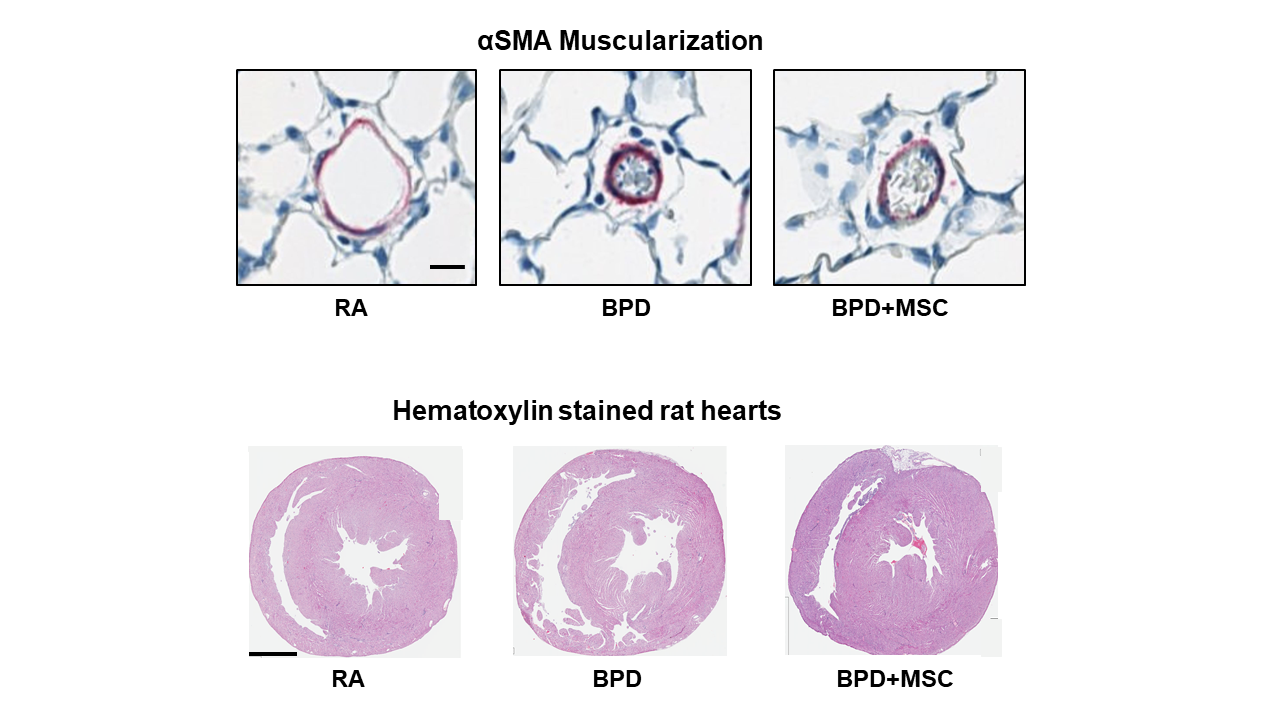

Supplement: Supplementary file 6 — Supplementary Figure 6: Alpha smooth muscle actin (SMA) staining of pulmonary blood vessels and hematoxylin stained hearts. No difference noted between the groups in pulmonary vessel muscularization nor right ventricle remodeling; n = all animals/group. RA = room air control; BPD = bronchopulmonary dysplasia; BPD + MSC = bronchopulmonary dysplasia treated with mesenchymal stomal cells. Scale bar for SMA = 10 μm and heart sections = 200 μm. [file SCT3-9-221-s006.tif]

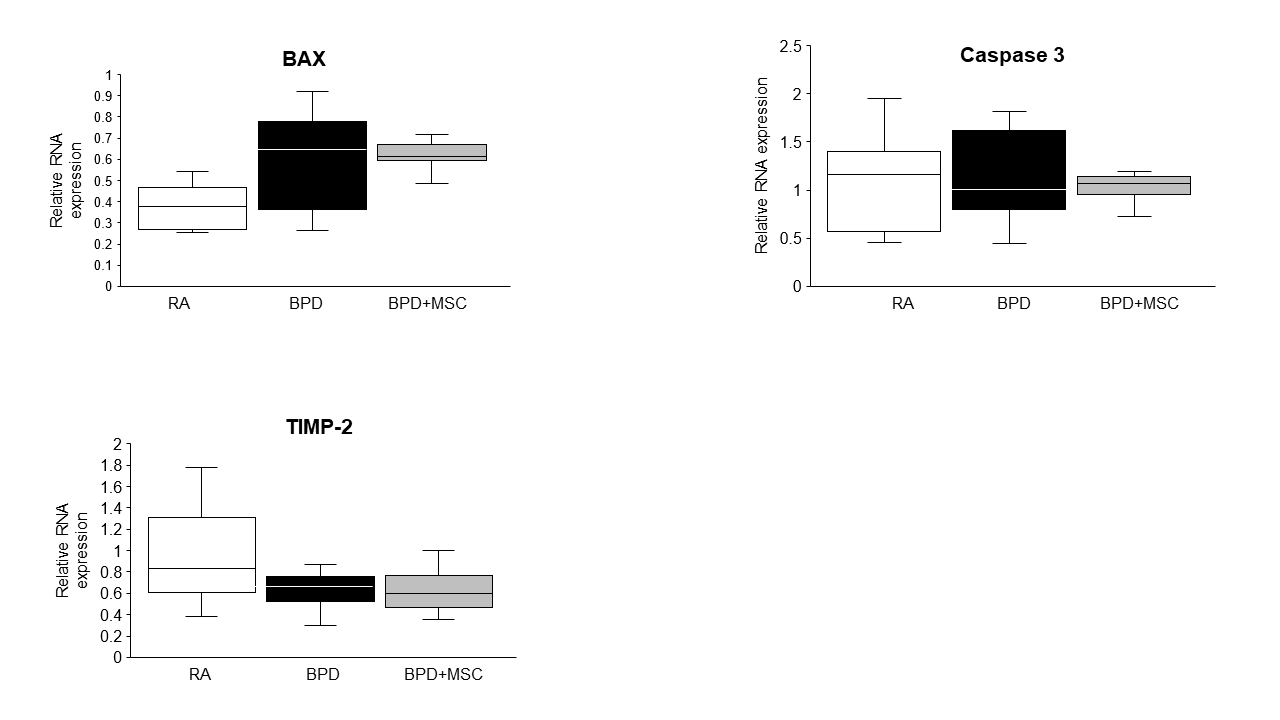

Supplement: Supplementary file 7 — Supplementary Figure 7: RT‐PCR data of rat lung homogenates. IL‐interleukin, TIMP‐tissue inhibitors of metalloproteinases, TGF‐transforming growth factor, VEGF‐vascular endothelial growth factor. Data are shown as median with IQR. RA = room air control; BPD = bronchopulmonary dysplasia; BPD + MSC = bronchopulmonary dysplasia treated with mesenchymal stomal cells. N = all animals/group. * P < 0.05 compared to RA. [file SCT3-9-221-s007.zip › SCT3_12626_Supplementary Figure 7 (bottom) R1.tif]

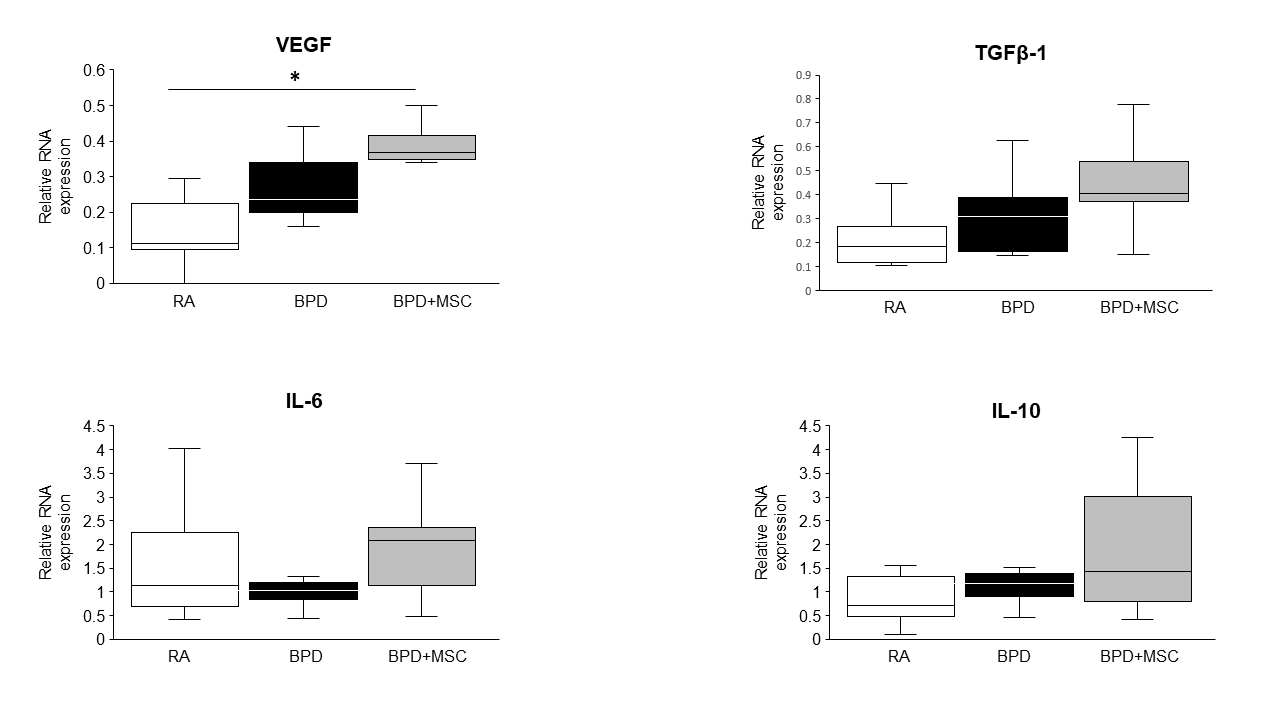

Supplement: Supplementary file 7 — Supplementary Figure 7: RT‐PCR data of rat lung homogenates. IL‐interleukin, TIMP‐tissue inhibitors of metalloproteinases, TGF‐transforming growth factor, VEGF‐vascular endothelial growth factor. Data are shown as median with IQR. RA = room air control; BPD = bronchopulmonary dysplasia; BPD + MSC = bronchopulmonary dysplasia treated with mesenchymal stomal cells. N = all animals/group. * P < 0.05 compared to RA. [file SCT3-9-221-s007.zip › SCT3_12626_Supplementary Figure 7 (top) R1.tif]
