# Supplementary material for: Intranasal delivery of human umbilical cord Wharton's jelly mesenchymal stromal cells restores lung alveolarization and vascularization in experimental bronchopulmonary dysplasia
Source: Stem Cells Transl Med. 2019 Nov 27;9(2):221–34. doi: 10.1002/sctm.18-0273 (PMC6988765; doi:10.1002/sctm.18-0273)
Supplement: Supplementary file 8 — Supplementary Figure 8: Sample protein array membranes for room air (RA) control, hyperoxia‐induced lung injury (BPD), and BPD animals treated with mesenchymal stromal cells (BPD + MSCs). (A) blots (B) all plots (C) statistical methodology for processes. Labels below were taken from vendor (R&D) pamphlet. [file SCT3-9-221-s008.docx]

**Supplementary Figure 8. Sample protein array membranes for room air (RA) control, hyperoxia-induced lung injury (BPD), and BPD animals treated with mesenchymal stromal cells (BPD+MSCs).** (A) blots (B) all plots (C) statistical methodology for processes.

**A**

**
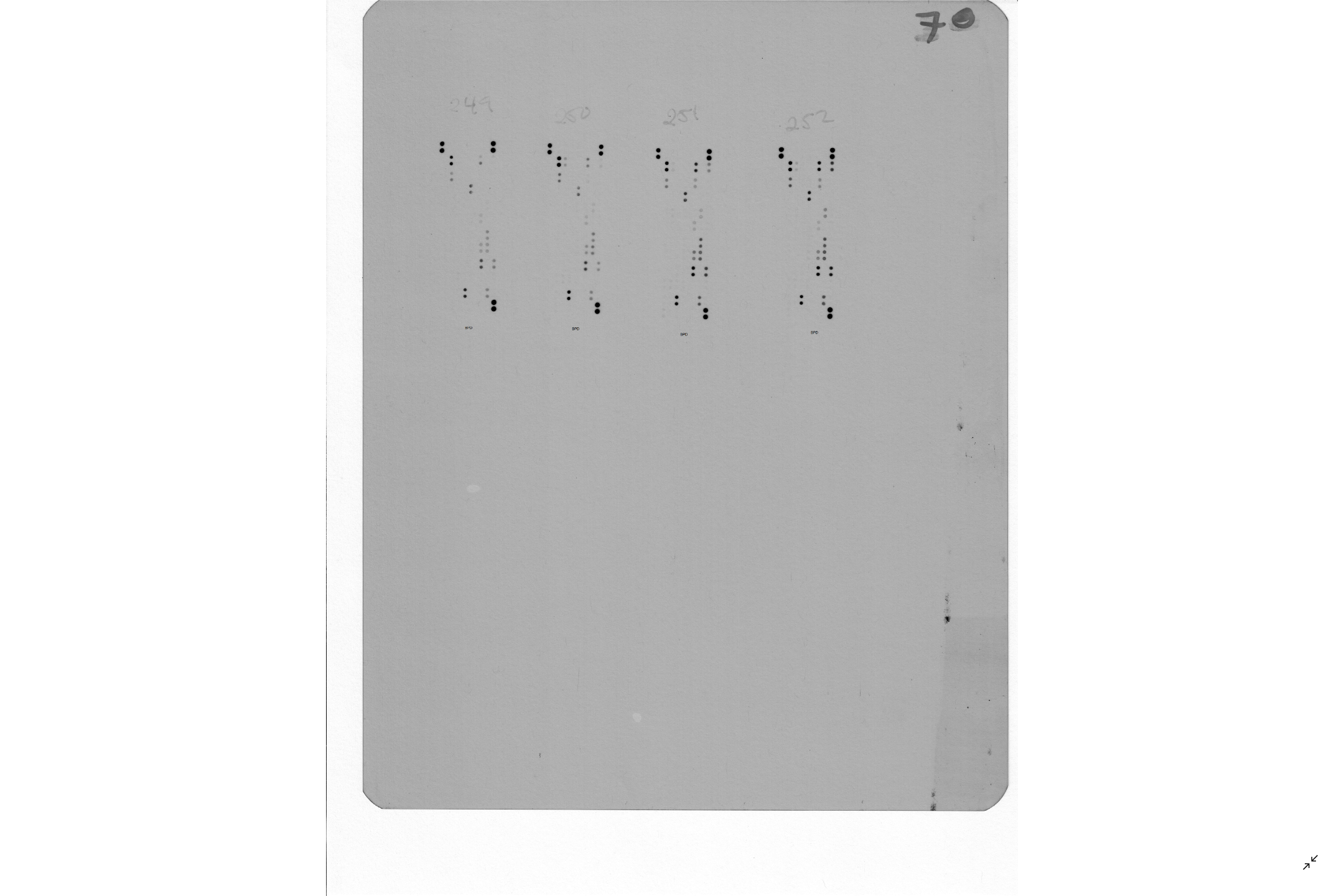
**


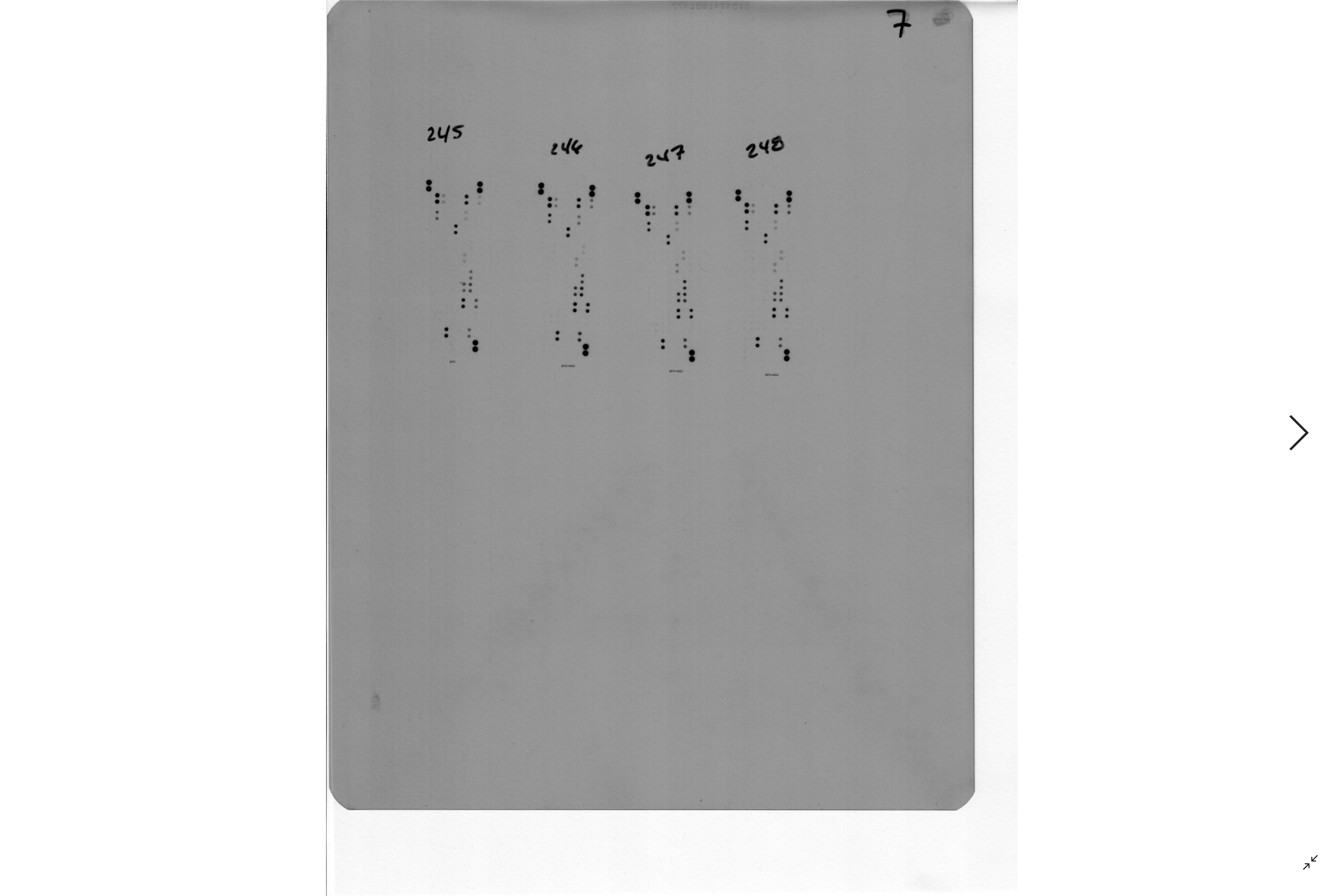


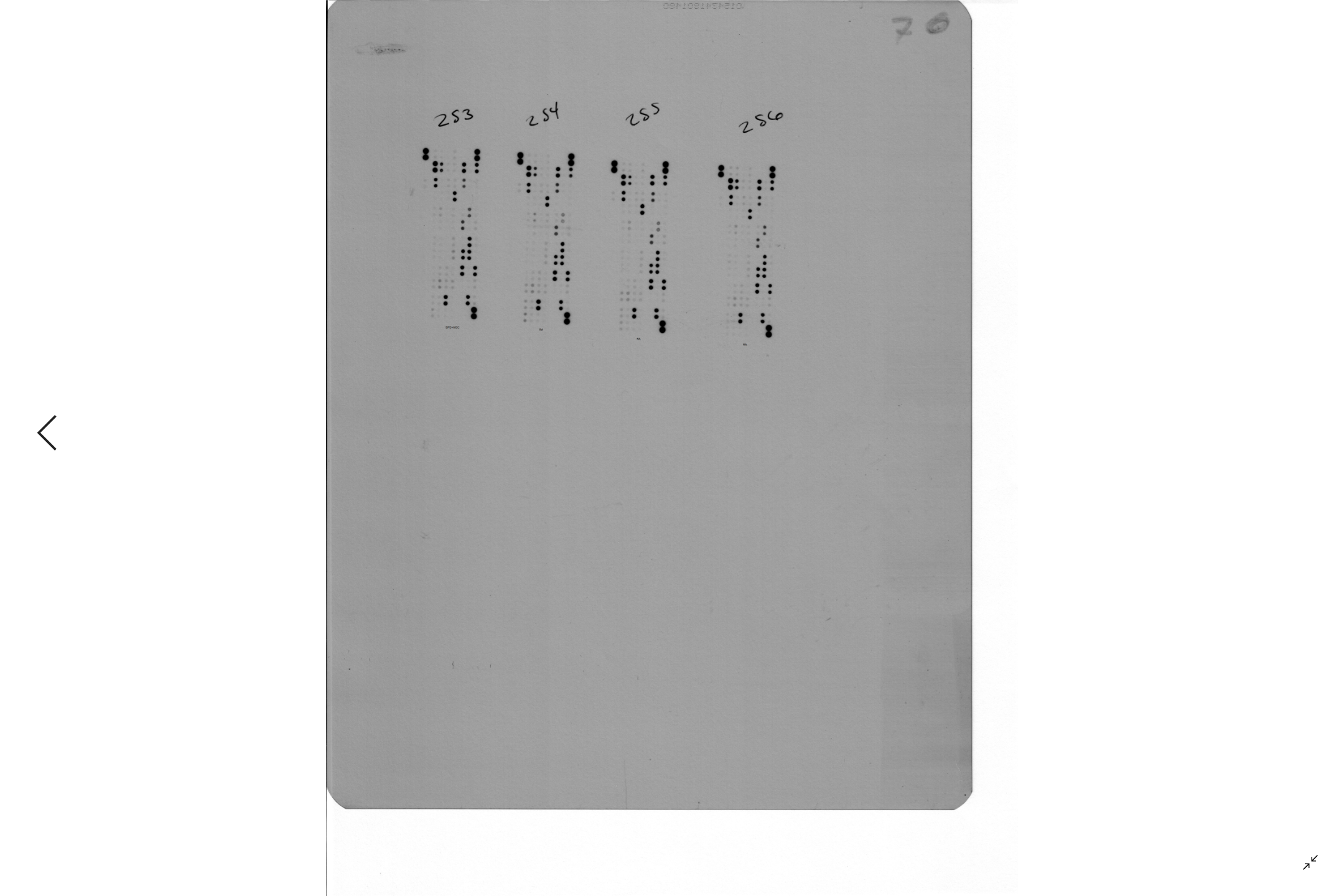
**B**


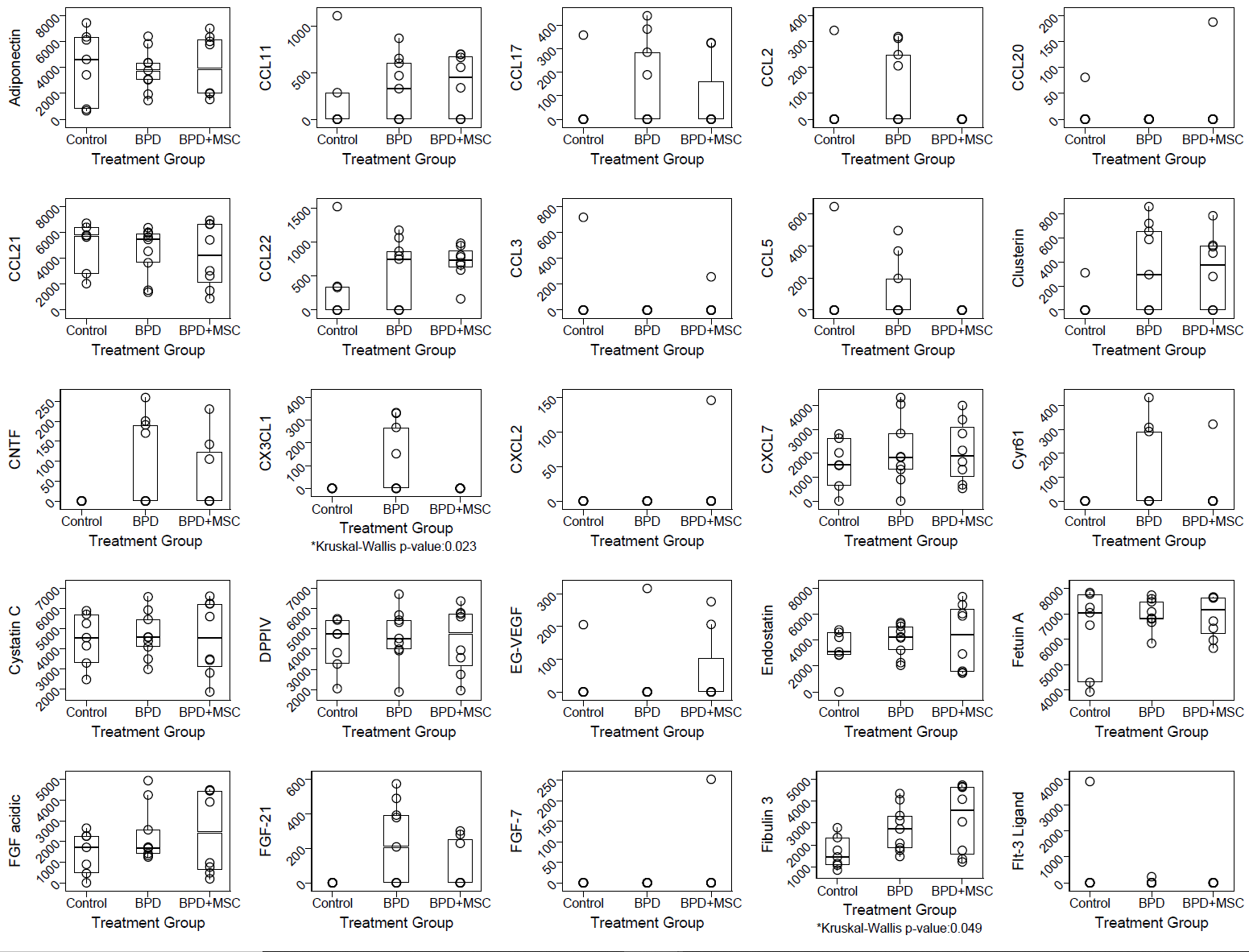


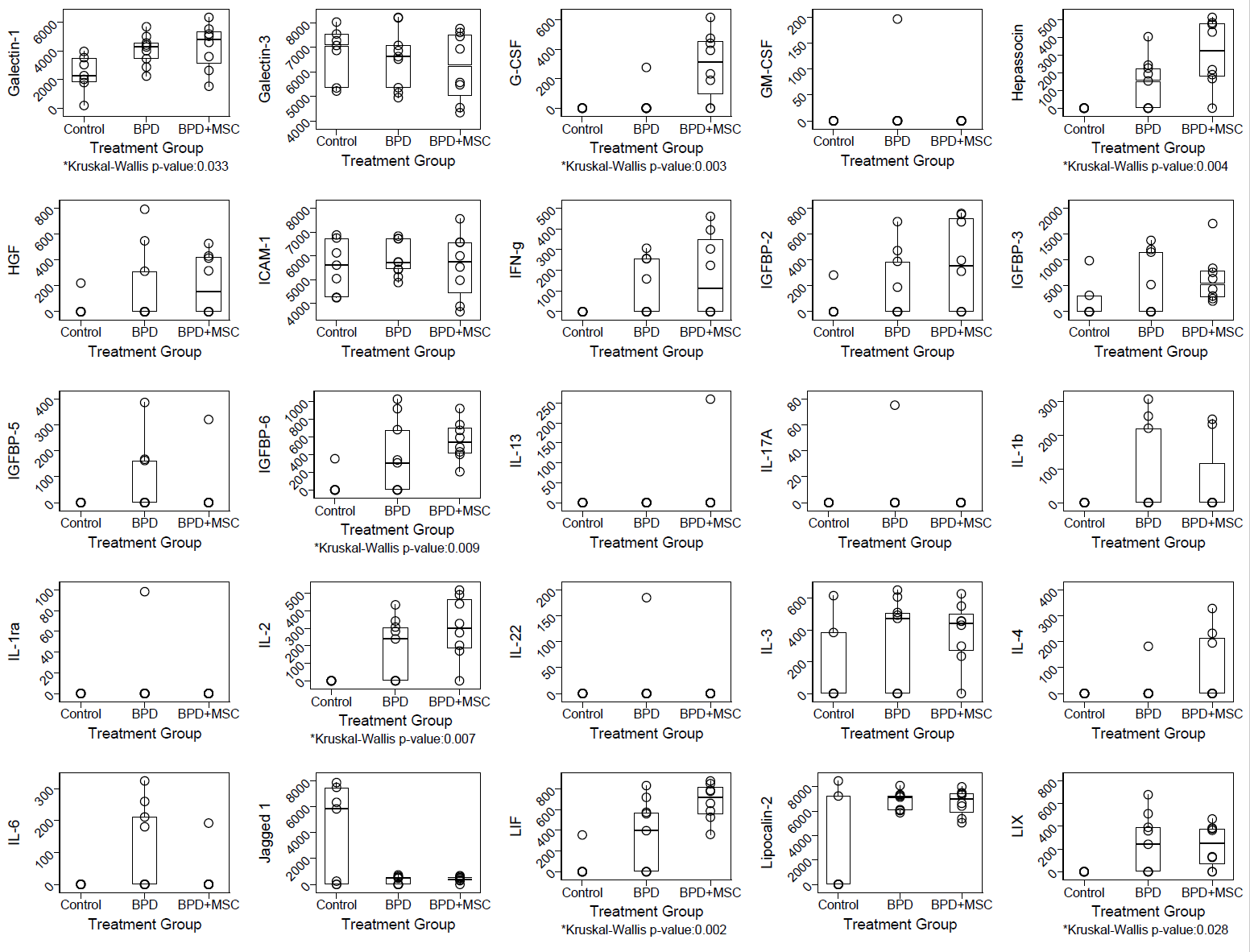


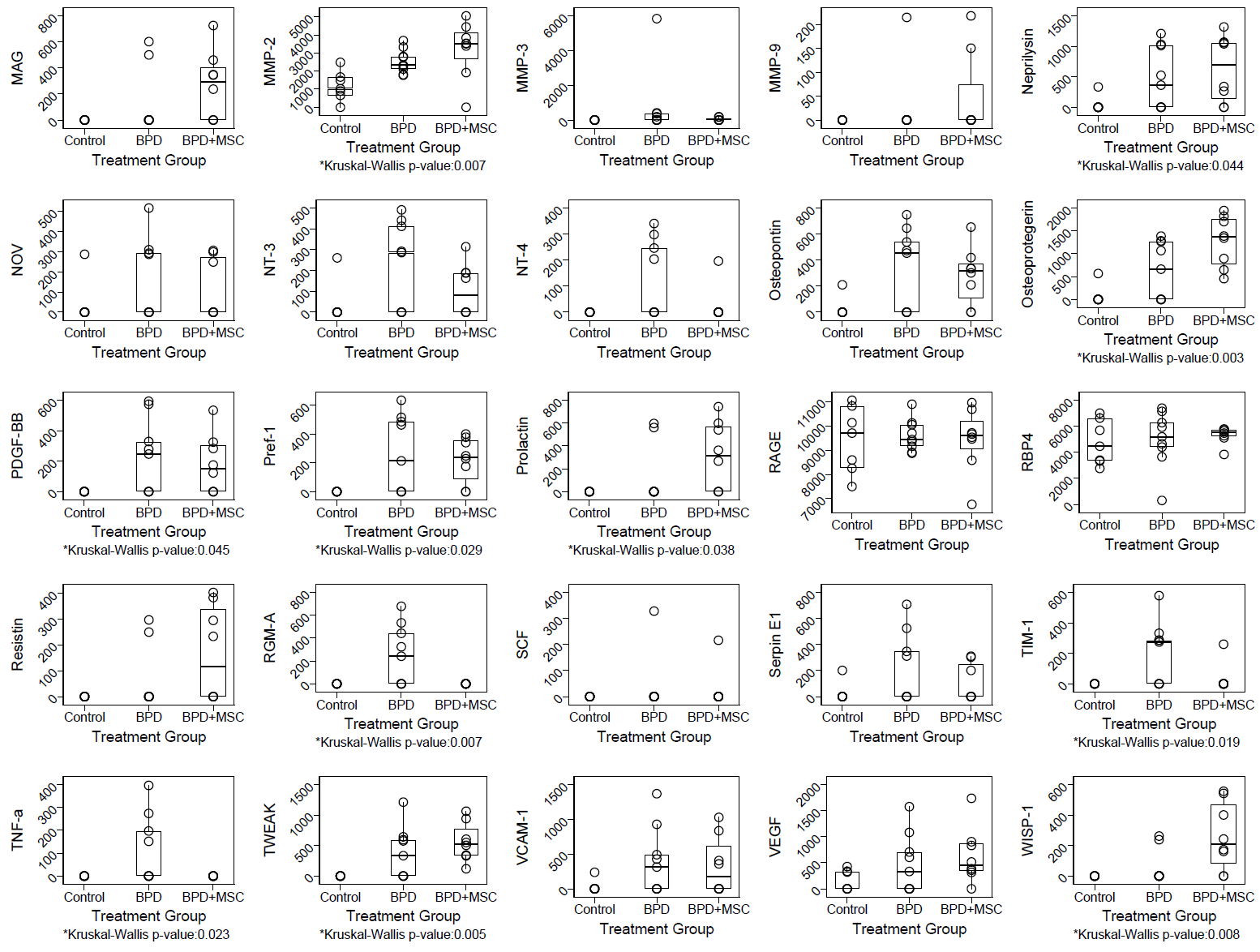


**C.**

Statistical analyses were performed in STATA version 16 (StataCorp LLC College Station, TX, USA). To assess differences in protein levels for the various cytokines examined, Kruskal-Wallis tests were performed followed by pairwise comparisons of the three treatment groups using Bonferroni’s adjusted significance level. Cytokines that were identified as significantly different by the Kruskal-Wallis test were subjected to additional analysis. Based on a search of the current literature, we identified the primary function of each cytokine. We then grouped the cytokines into six biological pathways: proinflammatory, anti-inflammatory, cell survival, wound healing, fibrogenesis, and anti-vascular. Scores for each pathway were generated by calculating the average of the standardized values for each cytokine within the pathway. The effect of the three treatment groups was then examined using ANOVA, again followed by pairwise comparisons using Bonferroni’s adjusted significance level.

Twenty cytokines were identified as significantly different by the Kruskal-Wallis test. Additionally, ten of these cytokines were further discriminated as significantly different between the control and MSC treated animals. Significant differences in mean protein content were also detected in all biological pathways. These results further confirm that our injury model was successful in generating a proinflammatory response, and more significantly, that MSCs may exert their reparative effects on the lung by modifying the inflammatory pathway.

StataCorp. 2019. Stata Statistical Software: Release 16. College Station, TX: StataCorp LLC.

Herve M. Caci, 1999. "KWALLIS2: Stata module to perform Kruskal-Wallis Test for equality of populations," Statistical Software Components S379201, Boston College Department of Economics, revised 20 Jun 1999.
