# Supplementary material for: Intranasal delivery of human umbilical cord Wharton's jelly mesenchymal stromal cells restores lung alveolarization and vascularization in experimental bronchopulmonary dysplasia
Source: Stem Cells Transl Med. 2019 Nov 27;9(2):221–34. doi: 10.1002/sctm.18-0273 (PMC6988765; doi:10.1002/sctm.18-0273)
Supplement: Supplementary file 9 — Supplementary Table 1 List of forward and reverse RNA primers. BAX = BCL2 associated x; Casp = caspase; IL = interleukin; TIMP = tissue inhibitor of metalloproteinases; VEGF = vascular endothelial growth factor; TGF = transforming growth factor. [file SCT3-9-221-s009.doc]

|  | **Sense** | **Antisense** |
| --- | --- | --- |
| **BAX** | AGGGTGGCTGGGAAGGC | TGAGCGAGGCGGTGAGG |
| **Casp3** | AGAGATTTGGTGCCACTATGAAT | CACTTGACATTATCGTTCCTATGC |
| **IL-6** | TAGTCCTTCCTACCCCAACTTCC | TTGGTCCTTAGCCACTCCTTC |
| **IL-10** | GTTGCCAAGCCTTGTCAGAAA | TTTCTGGGCCATGGTTCTCT |
| **TGFβ-1** | TACAGGGCTTTCGCTTCAGT | GTCCAGGCTCCAAATGTAGG |
| **TIMP2** | AAGGAGATGGCAAGATGCAC | TGTAGCATGGGATCATAGGG |
| **VEGF** | GGTTGCTCCTTCACTCCCTC | GTCTCTCTCTCTCTCTCTCTTCCTT |
| **β-Actin** | TACTCTGTGTGGATTGGTGGCTC | CATCGTACTCCTGCTTGCTGATAC |
