# Supplementary material for: Intranasal delivery of human umbilical cord Wharton's jelly mesenchymal stromal cells restores lung alveolarization and vascularization in experimental bronchopulmonary dysplasia
Source: Stem Cells Transl Med. 2019 Nov 27;9(2):221–34. doi: 10.1002/sctm.18-0273 (PMC6988765; doi:10.1002/sctm.18-0273)
Supplement: Supplementary file 10 — Supplementary Table 2 List of genes tested in the RT2 Profiler PCR Array for Rat Endothelial Cell Biology. [file SCT3-9-221-s010.doc]

| **Pathway** | **Genes** |
| --- | --- |
| **Angiogenesis** | Angpt1, Ccl2, Ccl5, Cx3cl1, Edn1, Ednra, Eng, F3, Faslg, Fgf1, Fgf2, Flt1, Fn1, Hif1a, Hmox1, Il1b, Il6, Itga5, Itgav, Itgb1, Itgb3, Kdr, Kit, Mmp2, Mmp9, Nos3, Nppb, Npr1, Pf4, Pgf, Plau, Ptgs2, Serpine1, Tek, Thbs1, Tymp, Vegfa, Xdh |
| **Vasoconstriction & Vasodilation** | Ace, Agt, Agtr1b, Apoe, Calca, Cav1, Cx3cl1, Edn1, Edn2, Ednra, F2r, Hmox1, Icam1, Nos3, Nppb, Npr1, Ptgis, Ptgs2, Sod1 |
| **Inflammatory Response** | Ace, Agt, Agtr1b, Apoe, Calca, Ccl2, Ccl5, Cx3cl1, Cxcl1, Cxcl2, Cxcr5, Ednra, F2r, F3, Fn1, Hif1a, Hmox1, Il1b, Il6, Nppb, Ptgs2, Sele, Selp, Tgfb1, Thbs1, Tnf, Vcam1 |
| **Apoptosis** | Anxa5, Bax, Bcl2, Bcl2l1, Casp1, Casp3, Cav1, Ccl2, Ccl5, Cflar, Cx3cl1, Edn1, Ednra, Fas, Faslg, Fgf2, Hif1a, Il1b, Il3, Il6, Il7, Ocln, Pf4, Ptk2, Tek, Thbs1, Tnf, Tnfsf10 |
| **Cell Adhesion Molecules** | Adam17, Agt, Bcl2, Calca, Cdh5, Col18a1, Cx3cl1, Eng, Fgf1, Fn1, Icam1, Il1b, Itga5, Itgav, Itgb1, Itgb3, Kdr, Pdgfra, Pecam1, Plau, Plg, Ptk2, Sele, Sell, Selp, Serpine1, Tgfb1, Thbs1, Tnf, Vcam1, Vegfa, Vwf |
| **Coagulation** | Anxa5, Cav1, Edn1, F2r, F3, Fn1, Mmp1, Pecam1, Pf4, Plat, Plau, Plg, Ptk2, Sell, Serpine1, Tek, Tfpi, Thbd, Thbs1, Timp1, Vwf |
| **Platelet Activation** | Apoe, Cx3cl1, F2r, Fn1, Il11, Il6, Itgb3, Nos3, Pdgfra, Pecam1, Pf4, Plg, Selp, Serpine1, Sod1, Tgfb1, Thbd, Thbs1, Timp1, Vegfa, Vwf |
