# Supplementary material for: Intranasal delivery of human umbilical cord Wharton's jelly mesenchymal stromal cells restores lung alveolarization and vascularization in experimental bronchopulmonary dysplasia
Source: Stem Cells Transl Med. 2019 Nov 27;9(2):221–34. doi: 10.1002/sctm.18-0273 (PMC6988765; doi:10.1002/sctm.18-0273)
Supplement: Supplementary file 11 — Supplementary Table 3 Advantages/disadvantages of the intranasal route for delivery of human umbilical cord mesenchymal stromal cells. [file SCT3-9-221-s011.docx]

| **ADVANTAGES** of intranasal delivery of human cord MSCs | **DISADVANTAGES** of intranasal delivery of human cord MSCs |
| --- | --- |
| Non-invasive | Biodistribution to the lungs may vary |
| Ease of administration | Nasal mucosa trauma/irritation |
| Straightforwardly provide multiple administrations | Onset of action dependent on bioavailability and nasal mucosal health |
| Local and systemic absorption | Relatively non-traditional delivery method |
| Avoids 1^st^ pass metabolism by liver | Device may be necessary to provide constant/reproducible dose |
| Sterile technique not necessary | Ciliary clearance |
| Readily available | May require carrier (ie, gel) to enhance delivery to lungs |
| Minimal pain | Enzymatic degradation in nasal cavity |
| Requires minimal training for administration |  |
| Convenient |  |
| Older population: compliance, self-delivery |  |
